# Host-Microbe Co-metabolism Dictates Cancer Drug Efficacy in *C. elegans*

**DOI:** 10.1016/j.cell.2017.03.040

**Published:** 2017-04-20

**Authors:** Timothy A. Scott, Leonor M. Quintaneiro, Povilas Norvaisas, Prudence P. Lui, Matthew P. Wilson, Kit-Yi Leung, Lucia Herrera-Dominguez, Sonia Sudiwala, Alberto Pessia, Peter T. Clayton, Kevin Bryson, Vidya Velagapudi, Philippa B. Mills, Athanasios Typas, Nicholas D.E. Greene, Filipe Cabreiro

**Affiliations:** 1 Institute of Structural and Molecular Biology, University College London and Birkbeck, London WC1E 6BT, UK; 2 UCL Great Ormond Street Institute of Child Health, University College London, London WC1N 1EH, UK; 3 Department of Computer Science, University College London, London WC1E 6BT, UK; 4 European Molecular Biology Laboratory (EMBL) Heidelberg, Genome Biology, Meyerhofstraße 1, 69117 Heidelberg, Germany; 5 Metabolomics Unit, Institute for Molecular Medicine Finland, University of Helsinki, 00290 Helsinki, Finland

**Keywords:** C. elegans, 5-FU, cancer, E. coli, Keio, chemical-genomics, autophagy, nucleotide metabolism, holobiont, co-metabolism

## Abstract

Fluoropyrimidines are the first-line treatment for colorectal cancer, but their efficacy is highly variable between patients. We queried whether gut microbes, a known source of inter-individual variability, impacted drug efficacy. Combining two tractable genetic models, the bacterium *E. coli* and the nematode *C. elegans*, we performed three-way high-throughput screens that unraveled the complexity underlying host-microbe-drug interactions. We report that microbes can bolster or suppress the effects of fluoropyrimidines through metabolic drug interconversion involving bacterial vitamin B_6_, B_9_, and ribonucleotide metabolism. Also, disturbances in bacterial deoxynucleotide pools amplify 5-FU-induced autophagy and cell death in host cells, an effect regulated by the nucleoside diphosphate kinase *ndk-1*. Our data suggest a two-way bacterial mediation of fluoropyrimidine effects on host metabolism, which contributes to drug efficacy. These findings highlight the potential therapeutic power of manipulating intestinal microbiota to ensure host metabolic health and treat disease.

## Highlights

• Drug-microbe-host high-throughput screens reveal new mechanisms for cancer drugs
• Microbes integrate nutritional and drug cues regulating treatment efficacy in the host
• Ribonucleotide co-metabolism of cancer pro-drugs exists between host and microbe
• Imbalanced bacterial deoxynucleotides synergize 5-FU-induced autophagic cell death

## Graphical Abstract

**Figure:**
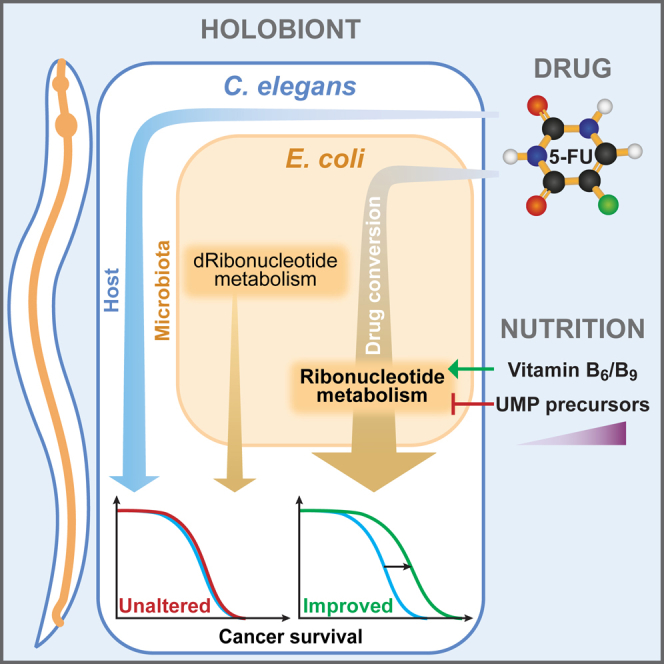

## Teaser

A three-way high-throughput screen involving host-microbe-drug interactions reveals that the beneficial impact of some drugs can be due to effects of drug-dependent alterations by gut microbe composition rather than direct action of the therapeutic itself.

## Introduction

Fluoropyrimidines are antimetabolite drugs primarily used to treat cancer. The archetype fluoropyrimidine, 5-fluorouracil (5-FU), is the principal therapy for colorectal cancer, the third most common cancer worldwide (Midgley and Kerr, 1999). 5-FU and its prodrugs, such as capecitabine, are uracil analogs that impede nucleotide biosynthesis, and hence cell division, by inhibiting thymidylate synthase (TS). Despite the widespread use of 5-FU-based chemotherapy, there is no universally accepted dose, and significant pharmacokinetic variations exist between individuals. As a result, adverse effects are frequent (diarrhea, nausea) and response rates sub-optimal (10% as monotherapy, 50% with combination therapies) (Longley et al., 2003). The observed inter-patient variation in 5-FU efficacy/toxicity has been attributed to several factors, including genetic polymorphisms in TS, dihydropyrimidine dehydrogenase, methylene THF reductase, and cytidine deaminase (Offer and Diasio, 2016). However, genetics alone does not explain differences in 5-FU tolerance between different continents suggesting environmental factors as key determinants in 5-FU action (O’Donnell and Dolan, 2009).

The influence of gut microbiota on 5-FU activity has not been explored. Gut microbes play an integral role in animal physiology, contributing to metabolism, influencing immunity, and modulating gut function. The thousands of species that make up the human microbiome surpass the host in terms of raw cell numbers, genomic diversity, and metabolic capability (Nicholson et al., 2012). Together, the host and its symbiotic microbiota (holobiont) act as a single evolutionary unit against environmental pressures (Rosenberg and Zilber-Rosenberg, 2011). Despite great advances in understanding the microbiome’s contribution to human health, one facet of the host-microbe relationship that remains undefined is the influence of microbiota on host-targeted drugs and the resulting contribution to host fitness. The consequences of not exploring these interactions at the holobiont level can be severe, as illustrated by the patient deaths due to the unanticipated microbial metabolism of the antiviral sorivudine to (E)-5-(2-bromovinyl)uracil, a suicide inhibitor of dihydropyrimidine dehydrogenase, a key host enzyme responsible for the detoxification of 5-FU (Okuda et al., 1997).

A significant barrier to exploring the host-microbiota relationship has been the complexity and diversity of the mammalian microbiome. The nematode *C. elegans* offers a simplified animal model with evolutionarily conserved features important for studying host-microbe interactions. Like humans, *C. elegans* have a symbiotic relationship with microbes, requiring them for nutrition, optimal development (Watson et al., 2014), and drug metabolism (Cabreiro et al., 2013).

Here, we employ an innovative host-microbe-drug model and high-throughput screening approaches to explore the role of microbes in modulating the effect of 5-FU and other fluoropyrimidines on *C. elegans*. We extensively define the microbial metabolic, genetic, and nutritional contributions to fluoropyrimidine activity on the host, revealing two distinct mechanisms by which bacteria modulate the efficacy of this class of anti-cancer drugs in vivo: (1) drug activation by bacterial ribonucleotide metabolism; and (2) influence of bacterial deoxynucleotide pools on the host response. We present a model that will be valuable for further probing host-microbe-drug interactions, highlighting how microbial metabolism can impact host fitness and survival in the context of pharmacotherapy.

## Results

### Microbes Have a Large Impact on Fluoropyrimidine Action in *C. elegans*

We reasoned that gut microbes may contribute to the activity of 5-FU in the context of the holobiont. Fluoropyrimidines impede cell division and interfere with nematode fertility and development, allowing simple and robust readouts of drug activity on the host, such as the minimum inhibitory concentration (MIC) for egg hatching (SenGupta et al., 2013).

**Figure 1:**
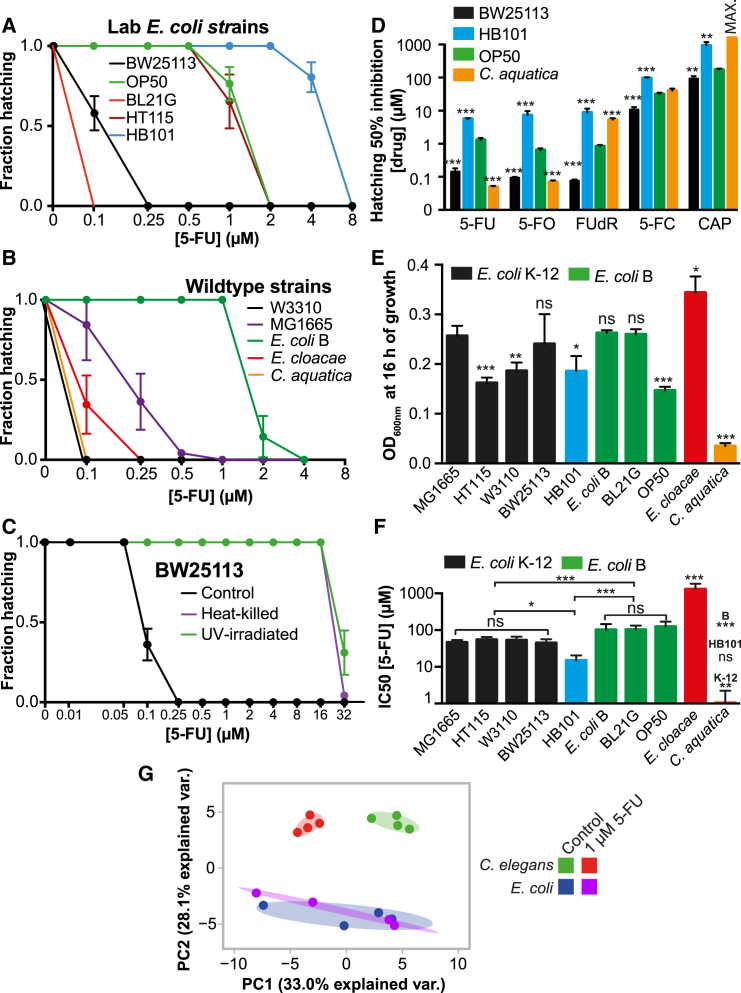
Bacterial Activity Modulates Fluoropyrimidine Efficacy in *C. elegans* (A and B) Worms cultured on laboratory (A) and WT (B) bacterial strains show disparate responses to 5-FU. *E. coli* K-12 strains: BW25113, HT115, W3310, MG1665; *E. coli* B strains: *E. coli* B, OP50, BL21G; K-12/B hybrid: HB101. (C) Heat/UV treatment of *E. coli* impairs 5-FU action. (D) Fluoropyrimidine effects on worms are bacterial strain specific. (E and F) Bacterial growth (E) and bacterial sensitivity to 5-FU (F) do not correlate with 5-FU effects in worms. (G) PCA of metabolomics data for *C. elegans* and *E. coli* treated with 5-FU. Data are represented as mean ± SD. ^∗^p < 0.05; ^∗∗^p < 0.01; ^∗∗∗^p < 0.001. See also Figure S1 and Table S1.

First, we compared 5-FU activity on worms fed five *Escherichia coli* laboratory strains commonly used in worm studies (Figure 1A) and observed up to 80-fold differences in 5-FU MICs (HB101, MIC = 8 μM versus BL21G, MIC = 0.1 μM, p < 0.001). The differences in host drug activity were independent of the bacterial strain serotype, as 5-FU efficacy was similarly variable between B strains (OP50 versus BL21G; 20-fold, p < 0.001) and within K-12 strains (BW25113 versus HT115; 8-fold, p < 0.001). This suggested that specific genotypic differences between the *E. coli* strains could be responsible for the observed variability in drug efficacy. We further explored other bacterial strains including the wild-type gut gammaproteobacteria *E. coli* B and K-12 strains (W3110 and MG1655), *Enterobacter cloacae* (LMG2783) and the soil betaproteobacterium *Comamonas aquatica* (DA1877) (Figure 1B). Again, large variations of 5-FU efficacy in worms were observed, with a 40-fold difference in egg hatching MIC between worms fed *C. aquatica* (0.1 μM) and *E. coli* B (4 μM, p < 0.001).

**Figure S1:**
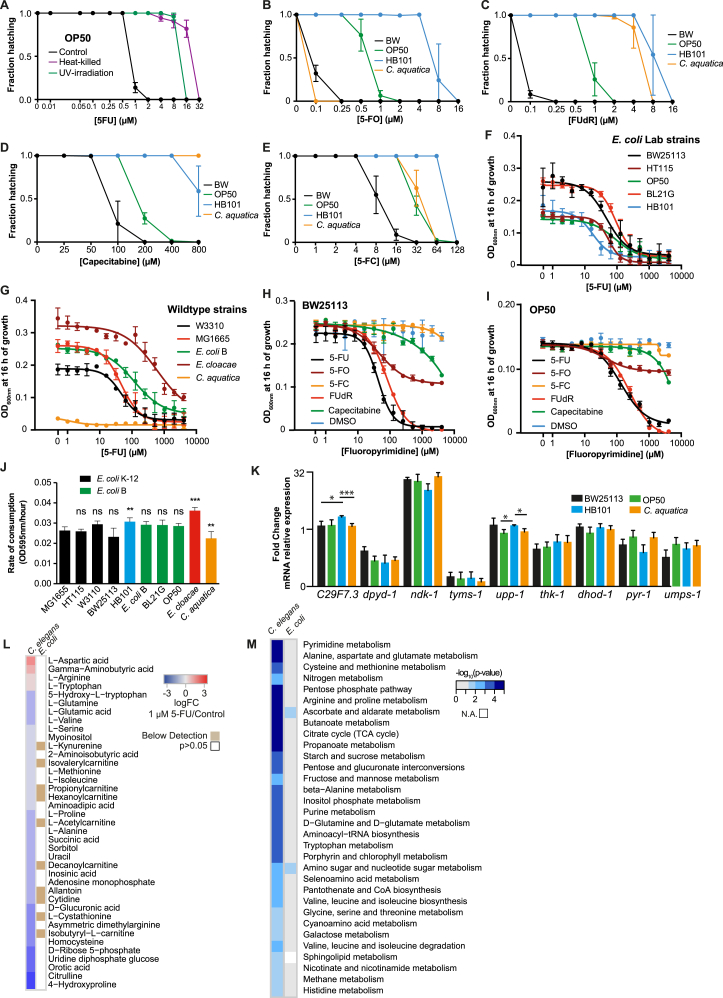
Effects of Fluoropyrimidines on Bacterial Growth and *C. elegans* Egg Hatching, Related to Figure 1 (A) *C. elegans* fed heat or UV-killed OP50 show increased resistance to 5-FU. MIC_Control_ = 2 μM; MIC_UV_ = 16 μM; MIC_Heat_ = 32 μM. (B–E) Fluoropyrimidines show varying efficacy on worms fed *E. coli* K-12/B hybrid HB101, K-12 BW25113, B OP50 and *Comamonas aquatica* DA1877. Response to 5-FO (B), FUdR (C), capecitabine (D) and 5-FC (E). (F and G) Effects of 5-FU on (F) laboratory and (G) wild-type bacterial growth in NGM broth. No significant changes in IC_50_ values were observed within bacterial strain serotype despite 8-fold changes in worm MICs (e.g., BW25113 versus HT115, Figures 1A, 2B, and 2F). Drug response curves were calculated using a log(inhibitor) versus response - variable slope (four parameter) model. *C. aquatica* grew poorly in NGM and was readily killed by 5-FU while *E. cloacae* grew the best on NGM with or without 5-FU (G), but both conferred low worm MICs (Figure 1B). (H and I) Effects of fluoropyrimidines on (H) BW25113 and (I) OP50 bacterial growth in NGM broth. Note that *E. coli* is remarkably resistant to growth inhibition by 5-FC but capable of modulating the pharmacodynamics of 5-FC in the worm hatching assays (E). (J) Rates of consumption of diverse bacterial strains by *C. elegans* over a period of 8 hr. Differences in bacterial consumption do not correlate with 5-FU efficacy. (K) Expression of worm genes involved in fluoropyrimidine metabolism does not correlate with bacterial-induced changes in drug efficacy. For example, increases in uridine monophosphate kinase (*C29F7.3*) and uridine phosphorylase (*upp-1*) expression for HB101-fed nematodes does not correlate with the reduced drug efficacy observed for this strain (Figures S1B–S1E) compared to OP50, BW25113 and *C. aquatica*. (L) Metabolite profiling in worms and *E. coli,* treated with 5-FU. BLOD = below level of detection. Only statistically significant changes in metabolite levels are displayed. (M) KEGG pathway enrichment analysis for metabolomics in *E. coli* and *C. elegans*. Metabolite concentration comparisons were made against control conditions in appropriate species. Grey indicates that enrichment is non-significant (p > 0.05), white – enrichment could not be estimated. Data are represented as mean ± SD. ^∗^p < 0.05; ^∗∗^p < 0.01; ^∗∗∗^p < 0.001. For statistics see Table S1.

Interventions modifying bacterial metabolic activity can influence the effect of host-targeted drugs in *C. elegans* (Cabreiro et al., 2013). Accordingly, we cultured nematodes on bacteria killed by heat or UV treatment. 5-FU efficacy was greatly decreased in nematodes fed dead BW25113 (128-fold, Figure 1C) or OP50 (Figure S1A). Thus, bacterial enhancement of 5-FU activity on *C. elegans* requires live bacteria.

Next, we explored the microbial modulation of other clinically used fluoropyrimidines, including floxuridine (FUdR), flucytosine (5-FC), capecitabine (CAP), and 5-fluoroorotic acid (5-FO). Fluoropyrimidine efficacy in *C. elegans* increased in the order CAP < 5-FC < 5-FU = FUdR = 5-FO. Within each drug treatment, the efficacy was highly dependent on the bacterial strain fed to the worms (e.g., 5-FU OP50 = 2 μM versus *C. aquatica =* 0.1 μM, p < 0.001; FUdR OP50 = 2 μM versus *C. aquatica =* 8 μM, p < 0.001) (Figures 1D and S1B–S1E). Therefore, as for 5-FU, the pharmacodynamics of these drugs in the host are bacteria dependent and drug specific.

A possible confounding factor for the differential effects of bacteria on worm MIC could be the absolute number of bacterial cells available and their digestion. To test whether there was a link between bacterial growth phenotype and modulation of drug efficacy in *C. elegans*, we quantified bacterial growth in the absence (Figure 1E) and presence (Figures 1F, S1F, and S1G) of 5-FU, and found no link with drug efficacy in *C. elegans*. Experiments with other fluoropyrimidines confirmed these observations (Figures S1H and S1I). Similarly, rates of bacterial consumption by worms do not explain 5-FU efficacy (Figure S1J).

The type of bacteria fed to *C. elegans* has a significant impact on host biology, including gene expression and metabolism (Watson et al., 2014). Expression of worm genes associated with pyrimidine metabolism, required for potential 5-FU conversion, was largely unaffected by the bacterial strain (Figure S1K). Therefore, bacterial modulation of 5-FU efficacy in *C. elegans* occurs by means other than bacterial regulation of nematode gene expression. Given that 5-FU disrupts pyrimidine metabolism homeostasis (Ser et al., 2016), we also investigated whether drug treatment induces metabolic changes in *E. coli*, which could contribute to the observed effects in the worm. Targeted metabolomics in worms or *E. coli* exposed to 1 μM 5-FU (the full inhibitory concentration for worm hatching) revealed that 5-FU did not affect bacterial metabolism as shown by principal component analysis (PCA), while inducing a significant metabolic shift in the worms (Figures 1G and S1L–S1M). These data imply distinct sensitivities to 5-FU action between host and microbe and support a regulatory effect of bacteria on drug efficacy in the host that is independent of altered bacterial metabolism caused by drug treatment.

Overall, we found that bacteria are key determinants of fluoropyrimidine efficacy on host metabolism and embryonic survival.

### Host-Microbe-Drug Screens Reveal Bacterial Regulators of Fluoropyrimidine Action

**Figure 2:**
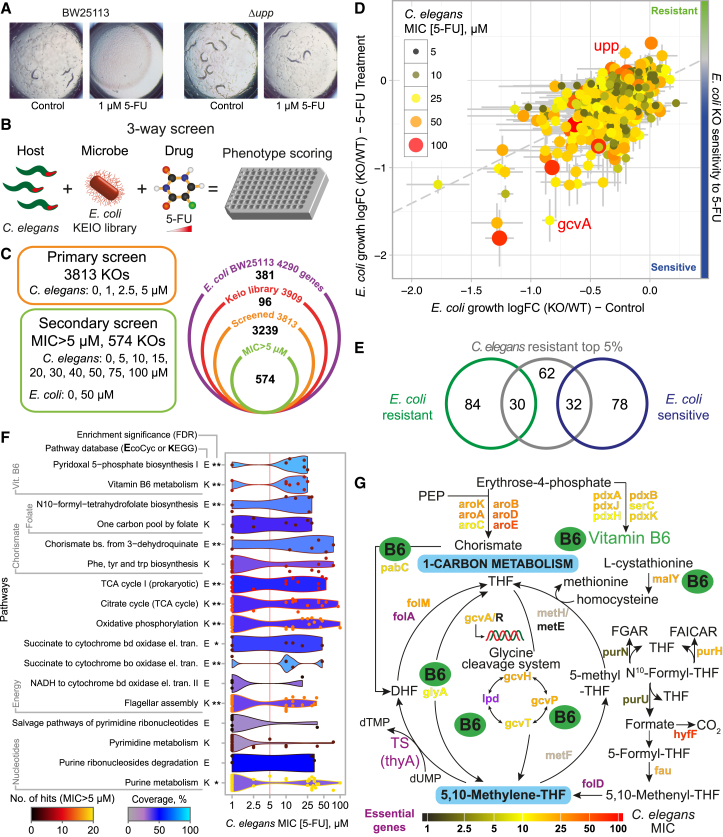
Chemical-Genomic Bacterial-Host Screens Identify Pathways for 5-FU Action Not Revealed by Bacterial-Drug Screens (A) 5-FU inhibits worm development on control (BW25113) but not mutant *E. coli* (Δ*upp*). (B) Diagram of the three-way host-microbe-drug interaction screen. (C) Screen design and Venn diagram of biologically relevant hits. (D) 3D graph correlating effects of gene knockout on bacterial growth (x axis), effects of 5-FU on bacterial growth (y axis), and effects of knockout on worm growth inhibition by 5-FU (colored circles). Gray dashed fit line (correlation between 5-FU and knockout effects in bacteria) determines bacterial sensitivity to 5-FU (blue/green color gradient box). Error bars represent SD. (E) Venn diagram of *E. coli* sensitive/resistant hits (FDR <0.05) with *C. elegans* 5% top hits for 5-FU treatment. (F) KEGG (K) and EcoCyc (E) pathway enrichment for gene deletions, and their effects on *C. elegans* ranked by coverage. Knockouts with MIC >5 μM are hits. Violin plots display distribution of MIC values; Contour color, number of hits; interior color, pathway coverage. ^∗^FDR <0.05; ^∗∗^FDR <0.01. (G) Metabolic network between chorismate, one-carbon, and vitamin B_6_ metabolism based on screen results. See also Figure S2 and Table S2.

**Figure S2:**
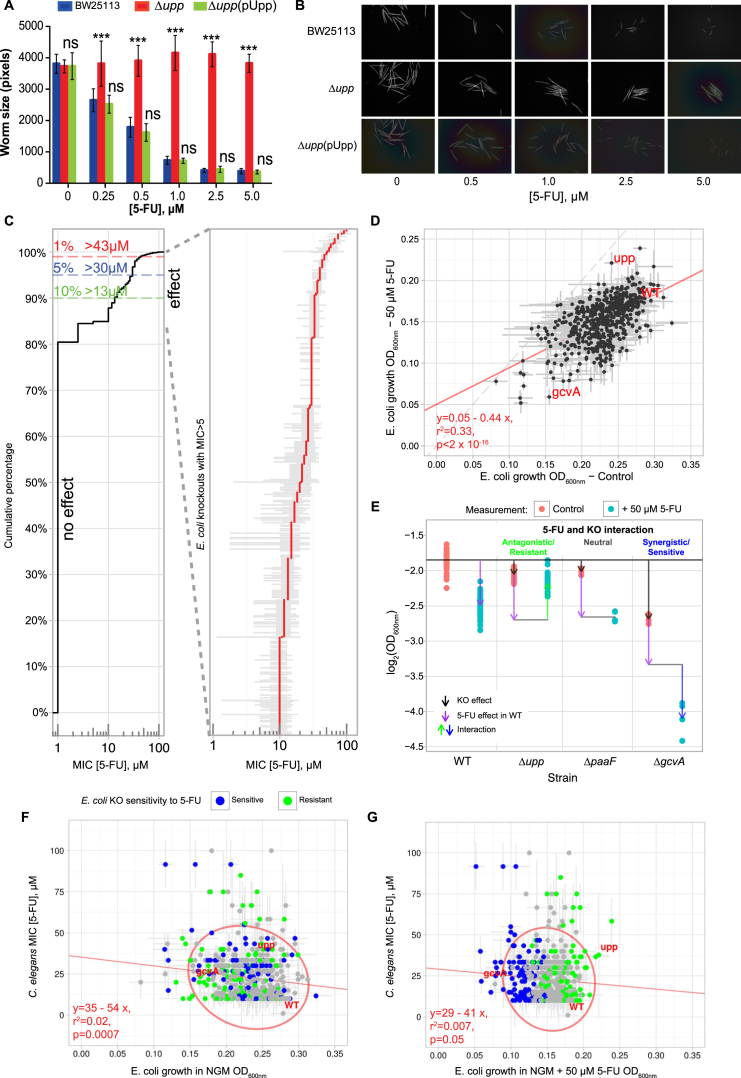
Chemical Genomic Bacterial Screens and Chemical Genomic Bacterial Host Screens, Related to Figure 2 (A) 5-FU inhibits development of worms fed control *E. coli* BW25113 but not *Δupp* mutants. This effect was reversed by gene complementation (pUpp). Worm size was obtained from fluorescence intensity measurements of the DA2123 worm strain. (B) Representative GFP fluorescence images of DA2123 worms from (A) illustrating the effects of 5-FU on worm size. (C) Cumulative distribution of *C. elegans* MIC values and variability (from 3 independent biological replicates) for 5-FU effects for each knockout with MIC > 5 μM. (D) Bacterial growth (OD_600nm_) of Keio knockouts in liquid NGM. Correlation between control and 50 μM 5-FU treatment. Linear fit (red) indicates general growth reduction by drug treatment (slope 0.44 < 1; p < 2 × 10^−16^). (E) Linear modeling applied for bacterial growth to determine antagonistic and synergistic hits in bacterial screen. Examples for WT, Δ*upp*, Δ*paaF* and Δ*gcvA*. Black arrows mark knockout effects in comparison to WT, purple arrows mark 5-FU treatment effect, gray bars indicate expected combined effect of knockout and 5-FU treatment. Significant interaction between knockout and 5-FU treatment is shown by green (antagonistic) and blue (synergistic) arrows. (F and G) Correlation between *C. elegans* MIC values and *E. coli* growth OD_600nm_ in NGM (F) or NGM + 50 μM 5-FU (G). Linear regression fit and covariation ellipse are marked in red. Knockouts that exhibit significant interaction with 5-FU (FDR < 0.05) are color-coded green (resistant) and blue (sensitive). Data are represented as mean ± SD. ^∗^p < 0.05; ^∗∗^p < 0.01; ^∗∗∗^p < 0.001. For statistics, see Table S2.

We hypothesized that the bacterial uracil phosphoribosyltransferase *upp* mutant (Δ*upp*), a gene previously used for cancer therapy (Koyama et al., 2000) and capable of metabolizing 5-FU in vitro, could influence the response of the host to treatment. As predicted, worms fed Δ*upp* bacteria developed fully at concentrations of 5-FU that completely inhibited worms fed the control strain (Figure 2A), further suggesting an interspecies mediation of drug effects. Therefore, to investigate how *E. coli* impacts 5-FU action in *C. elegans*, we developed a three-way high-throughput host-microbe-drug screen using the *E. coli* non-essential gene knockout Keio library (Baba et al., 2006) (Figure 2B). The developmental response of *C. elegans* to 5-FU is dose dependent when fed control BW25113 (Figures S2A and S2B). Therefore, the worm response to the drug in the presence of each *E. coli* mutant was estimated as the MIC that arrests development at L1 larvae stage, allowing us to determine the relative contribution of each *E. coli* gene in mediating 5-FU effects in the host. 96 mutants were excluded from further investigation due to poor growth on nematode growth medium (NGM), undisrupted target gene, or impaired worm development in the absence of drug (Figure 2C; Table S2). A primary screen within a narrow range of drug concentrations (1–5 μM) revealed that ∼80% of the total 3,813 *E. coli* mutants screened did not alter 5-FU effects in *C. elegans* (Figures 2C and S2C). 574 (15%) of the mutant strains allowed full development at 5 μM and were investigated further at a higher range of concentrations (5–100 μM). This secondary screen (Figures S2C) revealed that the top 5% (124 mutants) increased *C. elegans* resistance to drug to MICs >30 μM and the top 1% (35 mutants) to MICs >43 μM. Thus, we identified the subset of bacterial genes most significantly affecting 5-FU efficacy in the host.

Chemical perturbations in bacteria have been used to associate genes with phenotypes and function (Nichols et al., 2011). We therefore asked whether we could infer drug action on the host by evaluating the effect of 5-FU on bacteria alone. First, we investigated how gene deletions affect *E. coli* response to 5-FU, using changes in growth, measured by optical density (OD), as a readout of drug effects on the bacteria (Figures S2D and S2E). As expected, most deletions did not affect response to 5-FU treatment (r^2^ = 0.33, p < 2 × 10^−16^; Figure S2D). One of the most significant resistant hits was Δ*upp* (p < 0.001, false discovery rate [FDR] = 0, Figures 2D, S2E, and S2G). These findings validate our bacterial screening approach (see STAR Methods for details). Next, we asked whether the effects observed for bacterial growth matched the effects of 5-FU in the worms. Worm MIC values (colored circles) were plotted relative to the bacterial growth OD values with (y axis) and without (x axis) 5-FU treatment for each *E. coli* knockout strain (Figures 2D, S2F, and S2G). Strikingly, bacterial growth in control (r^2^ = 0.02, p = 0.0007; Figure S2F) or 5-FU treatment alone (r^2^ = 0.007, p = 0.05; Figure S2G) cannot predict 5-FU effects on host physiology. Moreover, when the three datasets are combined together (Figure 2D), this can be further inferred visually by the lack of a gradient in color top to bottom (correlation with 5-FU effects on bacterial growth) or left to right (correlation with bacterial growth alone). Indeed, the 5% of bacterial knockouts that conferred the greatest MIC values in worms contained equal proportions of mutants with sensitive (e.g., Δ*gcvA*) and resistant (e.g., Δ*upp*) growth interactions with 5-FU (Figure 2E). Altogether, bacterial growth or 5-FU effects on *E. coli* growth are not key determinants or predictors of 5-FU effects on host physiology.

To determine which bacterial processes contribute to the effects of 5-FU on host physiology, we performed enrichment analysis of KEGG and EcoCyc pathways on the genes with strong 5-FU effects on *C. elegans* development (MIC >5 μM) (Figure 2F, see STAR Methods for details). Out of the significantly enriched pathways, 3 (pyridoxal-5-phosphate [PLP], chorismate, and folate biosynthesis) were involved in the production of 5,10-methylene tetrahydrofolate (CH_2_-THF), a cofactor for TS (*thyA*/TS; Figure 2G), the canonical target of 5-FU (Longley et al., 2003). Enrichment for pathways involved in energy production (e.g., TCA cycle, oxidative phosphorylation) is consistent with the observation that disruption of metabolic activity by UV/heat treatment decreases 5-FU efficacy (Figures 1C and S1A). Surprisingly, pyrimidine metabolism was not significantly over-represented, suggesting that only key genes (e.g., Δ*upp*) belonging to this pathway contribute to the observed effects. Overall, our three-way screen reveals that host-microbe-drug interactions are inherently complex, and that drug efficacy at the holobiont level can only be fully understood if evaluating biological readouts in the host.

### *E. coli* Vitamin B_6_ Is Essential for 5-FU Efficacy in *C. elegans*

**Figure 3:**
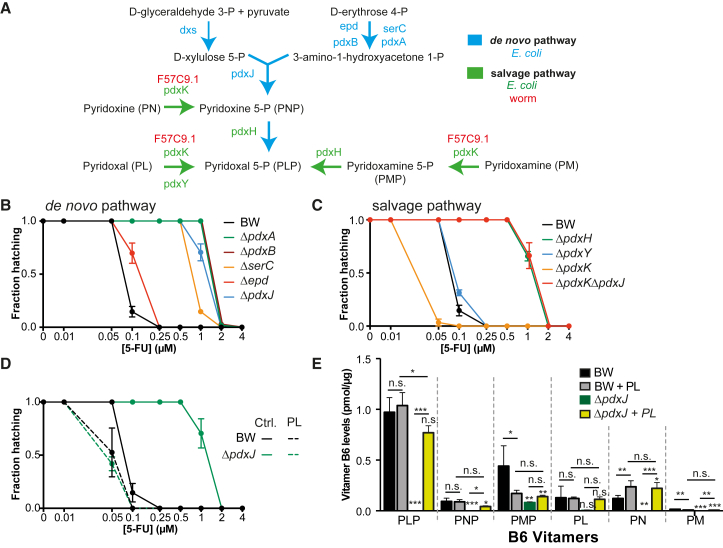
Pyridoxal-5-Phosphate Is a Key Cofactor for the Mediation of 5-FU Effects (A) The de novo (blue, *E. coli*) and salvage pathway genes (green, *E. coli*; red, *C. elegans*) of B_6_ metabolism. (B) Knockout of B_6_ de novo pathway enzymes in *E. coli* reduces 5-FU efficacy in worms. (C) *E. coli* B_6_ salvage pathway modulates the de novo pathway to regulate 5-FU effects on worms. (D and E) Supplementation of pyridoxal (PL) improves 5-FU efficacy in worms (D) and rescues bacterial B_6_ deficiency as measured by LC-MS/MS (E). Data are represented as mean ± SD. ^∗^p < 0.05; ^∗∗^p < 0.01; ^∗∗∗^p < 0.001. See also Figure S3. For statistics, see Table S3.

**Figure S3:**
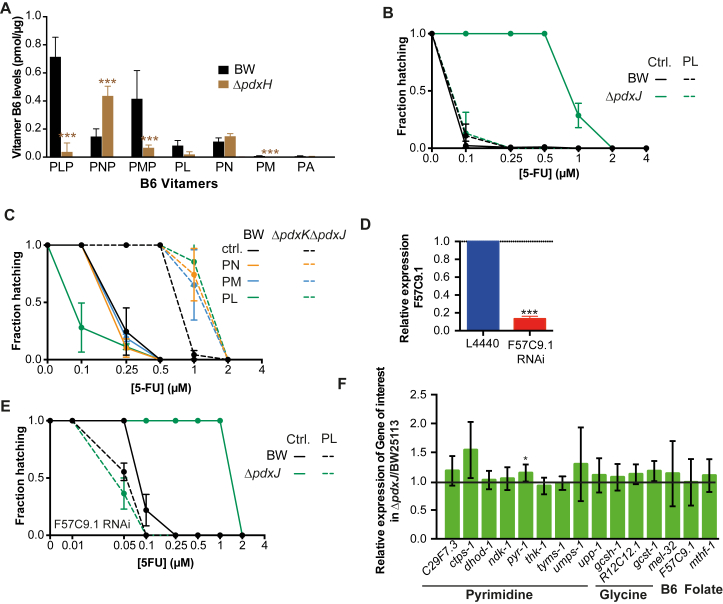
Effects of Bacterial Vitamin B_6_ Metabolism on 5-FU Efficacy, Related to Figure 3 (A) Vitamin B_6_ pools are altered in Δ*pdxH* mutant *E. coli* measured by LC-MS/MS. PLP = Pyridoxal-5-Phosphate; PNP = Pyridoxine-5-Phosphate; PMP = Pyridoxamine-5-Phosphate; PL = Pyridoxal; PN = Pyridoxine; PM = Pyridoxamine; PA = Pyridoxic acid. (B) Media supplementation with 10 μM PL rescues 5-FU efficacy on worms fed Δ*pdxJ* bacteria to control levels (BW + PL = 0.25 μM versus BW = 0.25 μM, p = 0.779; Δ*pdxJ* + PL = 0.25 μM versus Δ*pdxJ* = 2 μM, p < 0.001; Δ*pdxJ* + PL = 0.25 μM versus BW = 0.25 μM, p = 1; Δ*pdxJ* + PL = 0.1 μM versus BW + PL = 0.1 μM, p = 1). (C) Inhibition of both B_6_ de novo and salvage pathways using a Δ*pdxJ*Δ*pdxK* double mutant decreases 5-FU efficacy which cannot be rescued by supplementation with 1 mM PN, PM or PL. (D) Efficient RNAi knockdown of the worm *F57C9.1* gene, an ortholog of human PDXK. (E) Knockdown of *F57C9.1* by RNAi does not alter worm responses to 5-FU upon supplementation with PL. (F) Gene expression levels by qRT-PCR of enzymes involved in the glycine cleavage system, folate and vitamin B_6_ metabolism in worms grown on Δ*pdxJ* bacteria. Data are represented as mean ± SD. ^∗^p < 0.05; ^∗∗^p < 0.01; ^∗∗∗^p < 0.001. For statistics, see Table S3.

Given the link between vitamin B_6_, chorismate-folate, and pyrimidine metabolism, we next investigated how these bacterial pathways regulate 5-FU action in the host. Vitamin B_6_ is a key cofactor involved in numerous enzyme-catalyzed reactions in all living systems. Unlike eukaryotes, *E. coli* is capable of synthesizing PLP, the active form of B_6_, via both de novo and salvage biosynthetic pathways (Fitzpatrick et al., 2007) (Figure 3A). We therefore tested the impact of these two B_6_ pathways in regulating 5-FU efficacy in the host. We found that impairing de novo PLP synthesis decreased 5-FU efficacy in the worm by 8- to 16-fold (p < 0.001; Figure 3B). By contrast, deletion of *pdxY* from the salvage pathway had no effect (Figure 3C). Knockout of *pdxH*, a gene at the intersection of both pathways, decreased 5-FU efficacy by 8-fold (p < 0.001; Figure 3C). Using liquid chromatography-tandem mass spectrometry (LC-MS/MS), we found that Δ*pdxH* mutants were also deficient in PLP (–96%, p = 0.0001) while accumulating pyridoxine 5-P (PNP; +315%, p = 0.0001) (Figure S3A). Interestingly, knockout of *pdxK*, a key kinase in the salvage pathway, improved 5-FU efficacy (*ΔpdxK* = 0.1 μM versus BW25113 = 0.25 μM, p < 0.001; Figure 3C), possibly due to positive feedback into de novo synthesis of PLP. Indeed, when we blocked de novo PLP synthesis in the Δ*pdxK* by knocking out *pdxJ*, 5-FU efficacy dropped to that conferred by Δ*pdxJ* and Δ*pdxH* mutants alone (Figure 3C). Altogether, our data imply a key role of bacterial PLP in the mediation of 5-FU effects on host physiology.

Vitamins are important regulators of microbial metabolism but can also influence host metabolism. Therefore, we tested whether PLP affects bacterial or host metabolism to regulate 5-FU efficacy. Supplementation with 1 mM pyridoxal (PL), a precursor of PLP through conversion by pyridoxal kinase (bacterial = *pdxK* or worm = *F57C9.1*; Figure 3A), fully rescued the effect of the Δ*pdxJ* mutant and improved 5-FU efficacy (BW + PL = 0.1 μM versus BW = 0.25 μM, p = 0.006, Figure 3D). Similar effects were observed at 10 μM PL (Figure S3B). Also, supplementation with PL fully rescued PLP and pyridoxine (PN) levels in Δ*pdxJ* vitamin B_6_-deficient bacteria (Figure 3E). In contrast, worms fed Δ*pdxK*Δ*pdxJ* double mutants were not rescued by supplementation with various B_6_ precursors at 1 mM, consistent with the role of *pdxK* in the utilization of B_6_ vitamers (Figure S3C). In addition, the hatching inhibition caused by 5-FU in *C. elegans* treated with RNAi for pyridoxal kinase (*F57C9.1*) (Figures 3A and S3D), with reduced capacity to utilize B_6_ vitamers, was similar to wild-type (WT) worms when fed Δ*pdxJ* and supplemented with PL (Figures S3E and S3D), suggesting that PL rescue is not mediated by worm metabolism. Consistent with this idea, worms grown on the PLP-deficient mutant Δ*pdxJ* failed to show significant gene expression changes for enzymes involved in vitamin B_6,_ folate metabolism, the glycine cleavage system, and nucleotide metabolism, which mediate the effects of 5-FU in eukaryotic cells (Ser et al., 2016) (Figure S3F). Taken together, these results support a role for bacterial PLP in the modulation of drug efficacy in the host via effects on microbial metabolism rather than direct effects on the host.

### Vitamin B_6_ Regulates One-Carbon Metabolism in Bacteria to Mediate 5-FU Effects on the Host

One-carbon metabolism (OCM) plays an essential role in a myriad of biochemical processes. Out of the 43 enzymes in *E. coli* that require PLP as a cofactor, ten were found in our screen as regulating the effects of 5-FU on host physiology, with five belonging to OCM (Figures 2G). In particular, PLP is essential for reactions involved in the production of CH_2_-THF (Longley et al., 2003), an important cofactor in pyrimidine synthesis by TS.

**Figure 4:**
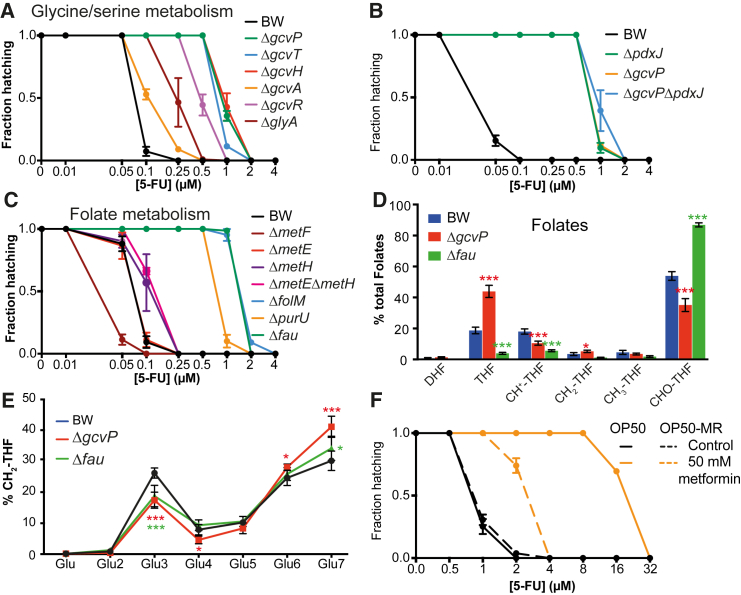
Vitamin B_6_ Acts in Concert with Glycine and Folate Metabolism to Mediate 5-FU Effects (A) Disruption of bacterial glycine and serine metabolism impairs 5-FU action. (B) B_6_ effects are mediated by the glycine cleavage system. (C and D) Disruption of bacterial folate metabolism (C) impairs 5-FU action in worms and alters folate homeostasis (D). DHF, dihydrofolate; THF, tetrahydrofolate; CH^+^-THF, 5,10-methenyl-THF; CH_2_-THF, 5,10-methylene-THF; CH_3_-THF, 5-methyl-THF; CHO-THF, 10-formyl-THF. Each metabolite is the ratio between the sum of the values for the different glutamate side chains (1–7) and the sum of all metabolites measured. (E) Disruption of glycine (Δ*gcvP*) or folate (Δ*fau*) metabolism alters CH_2_-THF polyglutamylation. (F) Metformin impairs 5-FU action in worms fed OP50 but to a lesser degree on metformin-resistant strain OP50-MR. Data are represented as mean ± SD. ^∗^p < 0.05; ^∗∗^p < 0.01; ^∗∗∗^p < 0.001. See also Figure S4. For statistics, see Table S4.

Glycine and serine metabolism are at the center of OCM, as major one-carbon donors in the production of CH_2_-THF (Locasale, 2013). We found that knockout of bacterial genes involved in the glycine cleavage system (GCS) (*gcvTHP*, or their transcriptional regulators *gcvA* and *gcvR*) reduced 5-FU effects up to 8-fold (Figure 4A). PLP is a key cofactor in the two parallel reactions mediated by *glyA* and *gcvP* to produce CH_2_-THF, and therefore we queried whether PLP regulates 5-FU effects via the GCS. Accordingly, host 5-FU MICs were similar for worms fed the single mutants, Δ*gcvP* and Δ*pdxJ*, and the respective double mutant (Figure 4B). These results support GCS as the main pathway regulated by PLP to mediate the efficacy of 5-FU.

**Figure S4:**
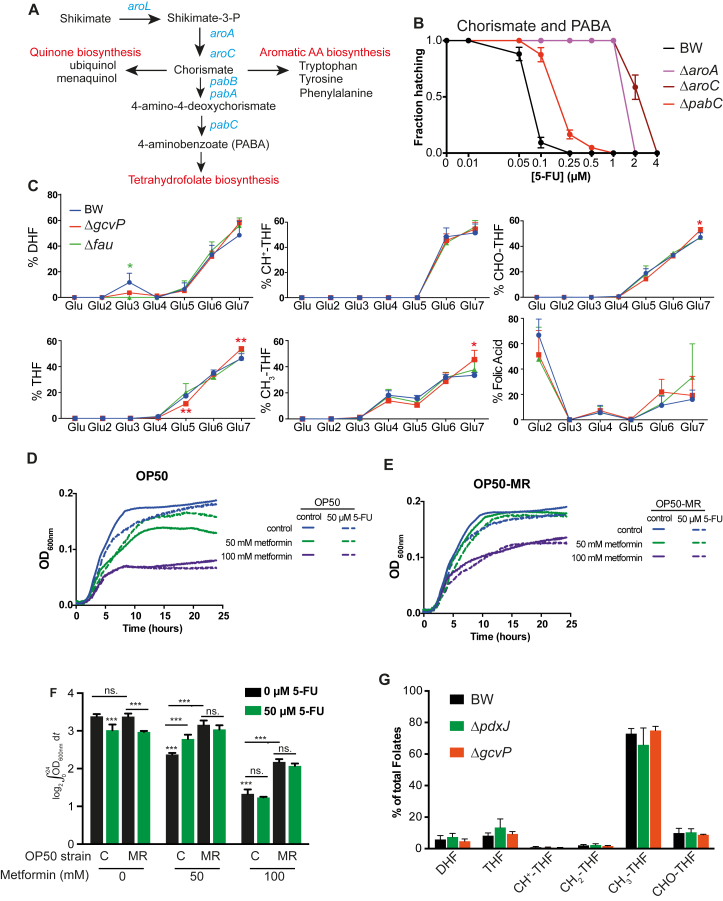
Bacterial Folate Metabolism Regulates 5-FU Action, Related to Figure 4 (A) Diagram of the chorismate and PABA biosynthetic pathways. (B) Disruption of chorismate and PABA biosynthesis impairs 5-FU action in worms. (C) Polyglutamylation profiles of detectable folate metabolites in control, Δ*gcvP* and Δ*fau* mutants. No striking changes are observed for the majority of folates. (D) 5-FU treatment (50 μM) impairs OP50 growth, but not in the presence of metformin (50 or 100 mM). (E) 5-FU treatment (50 μM) impairs OP50 metformin-resistant strain (OP50-MR) growth regardless of metformin effects (50 or 100 mM). (F) Metformin effects on bacterial growth are antagonistic to 5-FU effects in OP50 but not OP50-MR. (G) Impairment of the folate cycle (Δ*fau*) and the glycine cleavage system (Δ*gcvP*) in *E. coli* does not modulate folate metabolism in *C. elegans*. Each metabolite is the ratio between the sum of the values for the different glutamate side chains (1-7) and the sum of all metabolites measured. Data are represented as mean ± SD. ^∗^p < 0.05; ^∗∗^p < 0.01; ^∗∗∗^p < 0.001. For statistics, see Table S4.

Next, we probed the role of CH_2_-THF in mediating 5-FU effects. First, we tested whether the inhibition of bacterial folate metabolism alters 5-FU efficacy on the host. Inhibition of the shikimate pathway that produces the folate precursor para-aminobenzoic acid (PABA; Figure S4A) decreased 5-FU efficacy up to 16-fold (Figure S4B). Also, impairing synthesis of the main precursors of CH_2_-THF, including tetrahydrofolate (THF) by Δ*folM* or Δ*purU* and 5,10-methenyl-THF (CH^+^-THF) by Δ*fau*, reduced 5-FU efficacy by 8- to 16-fold (Figure 4C). In contrast, deletion of *metF* resulted in increased drug efficacy (MIC = 0.1 μM, p < 0.001; Figure 4C), possibly due to retention of CH_2_-THF within the folate cycle. Next, we performed LC-MS/MS analysis of bacterial folate metabolism in Δ*fau* and Δ*gcvP* mutants to investigate the impact of these genes on folate metabolism. Our data show a shift in folate metabolite pools with an increase in formyl-THF (CHO-THF; +33%, p < 0.0001) in Δ*fau*, and THF (+25%, p < 0.0001) in Δ*gcvP*, with a decrease in the remaining folate forms (Figure 4D), confirming their role in the regulation of folate metabolism and consistent with our observations in *Gldc*-deficient mouse embryos (Pai et al., 2015). These findings suggest a redistribution of folates to maintain essential CH_2_-THF pools required for cellular division. Altogether, the data indicate that bacterial folate metabolic status regulates the efficacy of 5-FU on the host.

In order to probe the activity of folate-dependent enzymes, we analyzed folate polyglutamylation profiles. Folates are defined by a pteridine ring, PABA, and a variable number of glutamate moieties, which determine retention and bioavailability for enzymatic reactions (Kwon et al., 2008). Major differences in polyglutamylation profiles were observed only for CH_2_-THF (Figure 4E) but not other folate forms (Figure S4C). The decrease in the abundance of shorter polyglutamate chains of CH_2_-THF in Δ*fau* (n_glu_ = 3) and Δ*gcvP* (n_glu =_ 3, n_glu =_ 4) (Figure 4E) is consistent with the role of these genes in the mediation of 5-FU efficacy in the host (Figures 4A–4C), possibly by regulating the availability of CH_2_-THF as a cofactor for TS. To confirm this, we used chemical interference by metformin, which dramatically alters polyglutamylation of CH_2_-THF in *E. coli* (Cabreiro et al., 2013). Our data show that metformin treatment impairs 5-FU treatment by 16-fold, and this effect is partially rescued in a strain resistant to metformin but not 5-FU (OP50-MR) (Figure 4F). Indeed, bacterial growth data show that metformin is antagonistic to the effects of 5-FU (Figures S4D–S4F). Given that genetic polymorphisms in human methylene THF reductase (Offer and Diasio, 2016) are associated with 5-FU efficacy in cancer treatment, we investigated the effect of Δ*gcvP* and Δ*pdxJ* mutants on host folate metabolism by LC-MS/MS. Our data show that these bacterial mutants do not regulate folate pools in the host (Figure S4G). Overall, our results indicate that disrupted folate metabolism in bacteria have a direct impact on 5-FU efficacy, without directly modulating host OCM.

### Fluoropyrimidines Hijack Bacterial Ribonucleotide Metabolism to Regulate Drug Efficacy on the Host

**Figure 5:**
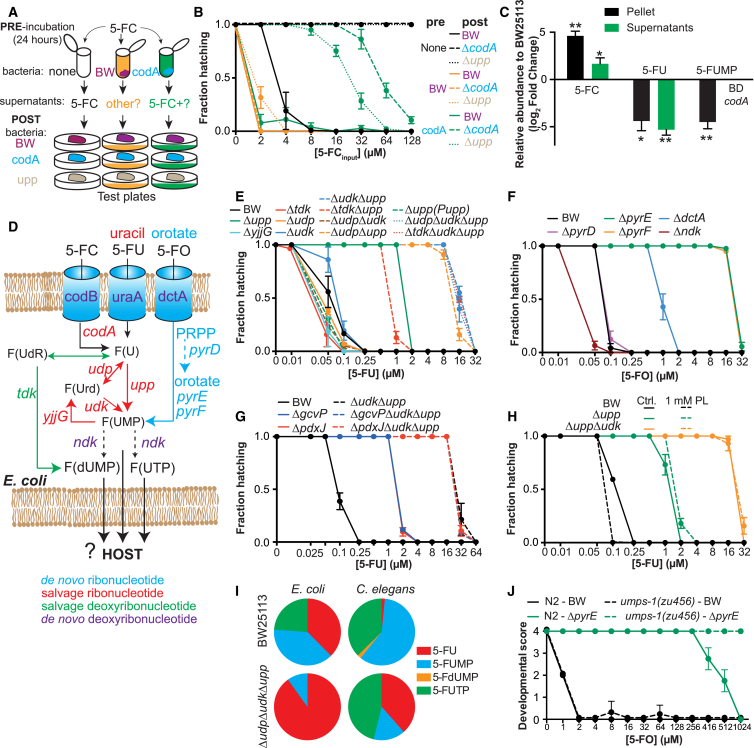
Bacteria Complement *C. elegans* Metabolically to Mediate 5-FU Effects (A) Diagram of the bioconversion experiment. (B) Pre-conversion of 5-FC by bacteria enhances drug effects on the host. (C) Control but not *codA* metabolize 5-FC and excrete 5-FU. BD-below detection in *codA* but not BW. (D) Diagram of bacterial (fluoro)pyrimidine metabolism. Dashed arrows, more than one reaction. (E) Opposite effects of salvage deoxyribonucleotide (Δ*tdk*) and ribonucleotide (Δ*upp*Δ*udk*Δ*udp*) metabolism in 5-FU efficacy. (F) Bacterial de novo pyrimidine metabolism regulates the effects of 5-FO. (G and H) B_6_ deficiency (G) and PL supplementation (H) regulate 5-FU effects through bacterial ribonucleotide metabolism. (I) Bacterial conversion of 5-FU alters fluoropyrimidine profiles and availability in *C. elegans.* (J) Knockout of host *umps-1* mediates drug effects on worm development only in the absence of bacterial conversion of 5-FO. Data are represented as mean ± SD. ^∗^p < 0.05; ^∗∗^p < 0.01; ^∗∗∗^p < 0.001. See also Figure S5. For statistics, see Table S5.

Fluoropyrimidines are pro-drugs that require intracellular metabolic conversion to downstream active metabolites to induce RNA and DNA damage (Longley et al., 2003). Since TS links OCM and pyrimidine metabolism, we hypothesized that bacteria could influence drug cytotoxicity through metabolic interconversion, an effect possibly regulated by vitamin B_6_. Cytosine deaminase (CodA), which converts cytosine to uracil, exists only in prokaryotes and has been used for suicide gene therapy against cancer for the delivery and conversion of 5-FC to 5-FU to cancer cells expressing bacterial *codA* (Koyama et al., 2000). We utilized this peculiarity to determine whether *E. coli* regulates drug efficacy independently of the host metabolic machinery by conducting a bioconversion assay (see STAR Methods for details, Figure 5A). 5-FC was effective at inhibiting worm hatching only if fed on WT bacteria but not Δ*codA* or Δ*upp* mutants (Figures 5A and 5B). Pre-incubation of 5-FC with control bacteria complemented the metabolic deficiency of Δ*codA* and Δ*upp*. Interestingly, the intermediate response observed for pre-incubation with Δ*codA* suggests an alternative but less efficient pathway for conversion of this pro-drug (Figure 5B). LC-MS/MS analysis of fluoronucleotides supports these findings (Figure 5C). The amount of 5-FC was higher in Δ*codA* relative to control (4.61-fold, p = 0.004; 1.7-fold, p = 0.044) with a concomitant decrease of 5-FU (4.4-fold, p = 0.018; 5.4-fold, p = 0.006) in pellets (intracellular) and supernatants (secreted), respectively. 5-fluorouridine 5′-monophosphate (5-FUMP) was also decreased in Δ*codA* pellets (4.5-fold, p = 0.0008). Thus, bacteria can convert and secrete metabolized fluoronucleotides from 5-FC, which then target worm metabolism (Figure 1G).

**Figure S5:**
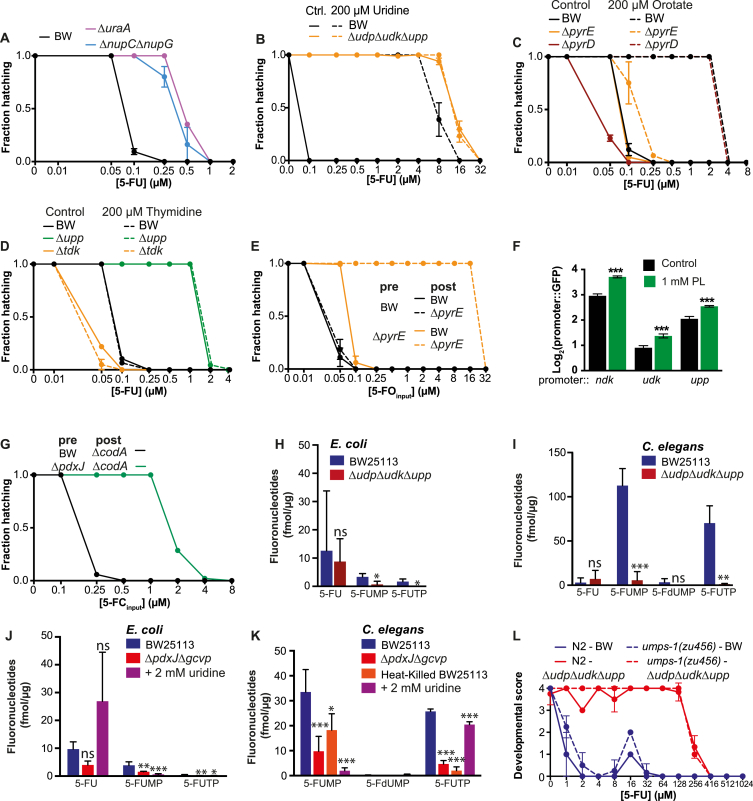
Bacterial Bioconversion of Fluoropyrimidines, Related to Figure 5 (A) Disruption of nucleobases (Δ*uraA*) and nucleosides import (Δ*nupC*Δ*nupG*) reduces 5-FU efficacy. (B) Supplementation with 200 μM uridine impairs 5-FU effects in worms fed BW25113 by 320-fold but not the triple mutant Δ*udp*Δ*udk*Δ*upp*. (C) Supplementation with 200 μM orotate impairs 5-FU effects in worms fed BW25113. The effect is partially rescued when worms are fed Δ*pyrE* but not Δ*pyrD* mutant bacteria. (D) Supplementation with 200 μM thymidine improves 5-FU effects in worms fed Δ*tdk* mutant bacteria but not control BW25113 or Δ*upp* mutants. (E) Pre-conversion of 5-FO by WT bacteria enhances drug effects on the host. Note that incubation of 5-FO with Δ*pyrE* mutant bacteria fully abolishes bacterial-mediated effects on host metabolism. Thus, de novo nucleotide metabolism is the unique pathway for the conversion of 5-FO. (F) Vitamin B_6_ supplementation increases GFP expression in *ndk, udk*, and *upp* promoter reporter strains. (G) Disruption of bacterial vitamin B_6_ production impairs 5-FC bioconversion. (H) LC-MS/MS quantification of fluoropyrimidines in *E. coli* supplemented with 50 μM 5-FU. (I) LC-MS/MS quantification of fluoropyrimidines in *C. elegans* supplemented with 50 μM 5-FU. (J) LC-MS/MS quantification of fluoropyrimidines in *E. coli* supplemented with 50 μM 5-FU. (K) LC-MS/MS quantification of fluoropyrimidines in *C. elegans* supplemented with 50 μM 5-FU. (L) Developmental response to 5-FU is uniquely mediated by bacterial genotype (BW control or triple mutant Δ*udp*Δ*udk*Δ*upp*) but not host genotype (N2 Wild-type or *umps-1(zu456)*). Data are represented as mean ± SD. ^∗^p < 0.05; ^∗∗^p < 0.01; ^∗∗∗^p < 0.001. For statistics, see Table S5.

Therefore, which bacterial genes are involved in the full bioconversion of fluoropyrimidines? To explore this, we first investigated the contribution of the salvage pathways of pyrimidine ribonucleotides (e.g., UMP) and deoxyribonucleotides (e.g., thymidine) (Figure 5D). Impeding bacterial uptake of nucleobases and nucleosides reduced 5-FU action (Figure S5A). Inhibition of ribonucleotide metabolism through knockout of *udp* and/or *udk* had no role alone (Δ*udp*Δ*udk* = 0.25 μM, p = 0.916) but acted synergistically with Δ*upp* to inhibit bacterial contribution of 5-FU effects on the host (Δ*upp =* 2 μM*;* Δ*udp*Δ*upp* = 32 μM; Δ*udk*Δ*upp* = 32 μM, Figure 5E). As expected, knockout of the pyrimidine nucleotidase *yjjG* improved drug efficacy (Δ*yjjG =* 0.1 μM, p = 0.014). We further investigated the link between pyrimidine supplementation, bacteria, and 5-FU effects on the host by feeding worms bacterial mutants of pyrimidine metabolism (Figure 5D). Supplementation of uridine (Figure S5B) and orotate (Figure S5C) dramatically reduced 5-FU efficacy (by 160- and 16-fold, respectively), an effect mediated by the ribonucleotide salvage pathway genes *upp,udk,udp* and the de novo pyrimidine pathway gene *pyrE*, but not *pyrD*, respectively (Figures S5B and S5C). Instead, interfering with the deoxyribonucleotide salvage pathway increased 5-FU efficacy by rewiring nucleotide metabolism though the ribonucleotide salvage pathway (Δ*tdk* = 0.1 μM, versus BW = 0.25 μM, p = 0.004; Δ*tdk*Δ*udp*Δ*upp* = 32 μM versus Δ*udk*Δ*upp* = 32 μM, p = 1), a finding consistent with the observation that supplementation with thymidine slightly increases 5-FU efficacy in worms fed Δ*tdk* bacteria (Figure S5D).

Next, we addressed the role of the de novo pathway. We observed that knockout of genes downstream (*pyrE* and *pyrF*) but not upstream (*pyrD*) of orotate strongly impaired 5-FO efficacy (Δ*pyrE* and Δ*pyrF* = 32 μM*;* Δ*pyrD* = 0.25 μM) (Figure 5F). In addition, bioconversion of 5-FO and excretion of metabolized fluoronucleotides can be performed by WT *E. coli* but not Δ*pyrE* (Figure S5E). We also found pathway specificity in the conversion of fluoronucleotides, as worms fed Δ*pyrE* did not modulate 5-FU effects (Figure S5C) or downstream metabolites of converted 5-FO (Figure S5E). Thus, bacterially mediated bioconversion and secretion of fluoropyrimidines is pathway specific (de novo for 5-FO and salvage ribonucleotide for 5-FU and 5-FC) and determines drug efficacy in the host. Furthermore, our data suggest that *E. coli* also acts as an integrator of nutritional metabolites to regulate the effects of fluoropyrimidines on host metabolism.

Therefore, we tested whether B_6_ and B_9_ act in concert with ribonucleotide metabolism to mediate drug transformation. Our data indicate that *pdxJ* and *gcvP* are negatively epistatic to *upp,udk* (Figure 5G). Also, supplementation of PL required intact ribonucleotide salvage to improve 5-FU efficacy (Figure 5H) and increased the transcription of pyrimidine metabolism genes (Figure S5F), and *pdxJ* regulates drug bioconversion (Figure S5G). Altogether our findings implicate this metabolic axis as central for fluoropyrimidine metabolism, and the regulation of nucleotide flux is mediated by vitamin B_6_ and B_9_.

Gut microbes are key players involved in the regulation of host physiology through metabolism of nutrients and xenobiotics (Spanogiannopoulos et al., 2016). Does co-metabolism of fluoropyrimidines occur in the worm-*E. coli* holobiont? We performed fluoronucleotide LC-MS/MS analysis of worms and *E. coli* treated with 5-FU. Our data highlight distinct profiles both in worms and bacteria, an effect dependent on *E. coli* genotype (Figure 5I). In particular, 5-FUMP and 5-fluorouridine 5′-triphosphate (5-FUTP) were significantly decreased in Δ*udp*Δ*udk*Δ*upp* triple mutants and worms fed this strain (Figures S5H and S5I). Similar trends were observed for worms fed heat-killed bacteria, Δ*pdxJ*Δ*gcvP* double mutants and control bacteria supplemented with uridine (Figures S5J and S5K), further supporting the role of bacterial ribonucleotide salvage metabolism in the conversion of fluoronucleotides to increase drug efficacy in the host. In order to probe this further, we utilized *umps-1(zu456)* loss-of-function worm mutants, which are incapable of converting orotate to UMP (Merry et al., 2014). Since *umps-1(zu456)* impairs fertility, we assayed worm larval development (which was not affected by the mutation; data not shown) as a proxy for drug efficacy. Indeed, *umps-1(zu456)* worms were only resistant to the effects of 5-FO when fed Δ*pyrE* bacteria (Figure 5J), but not to 5-FU (Figure S5L). Overall, our data suggest that host de novo pyrimidine metabolism does not contribute to fluoropyrimidine efficacy beyond its minor role in converting 5-FO at high drug concentrations and supports the notion that the holobiont functions as a single unit in the mediation of drug effects.

### Bacterial Deoxynucleotide Imbalance Increases 5-FU-Induced Autophagy and Cellular Death

**Figure 6:**
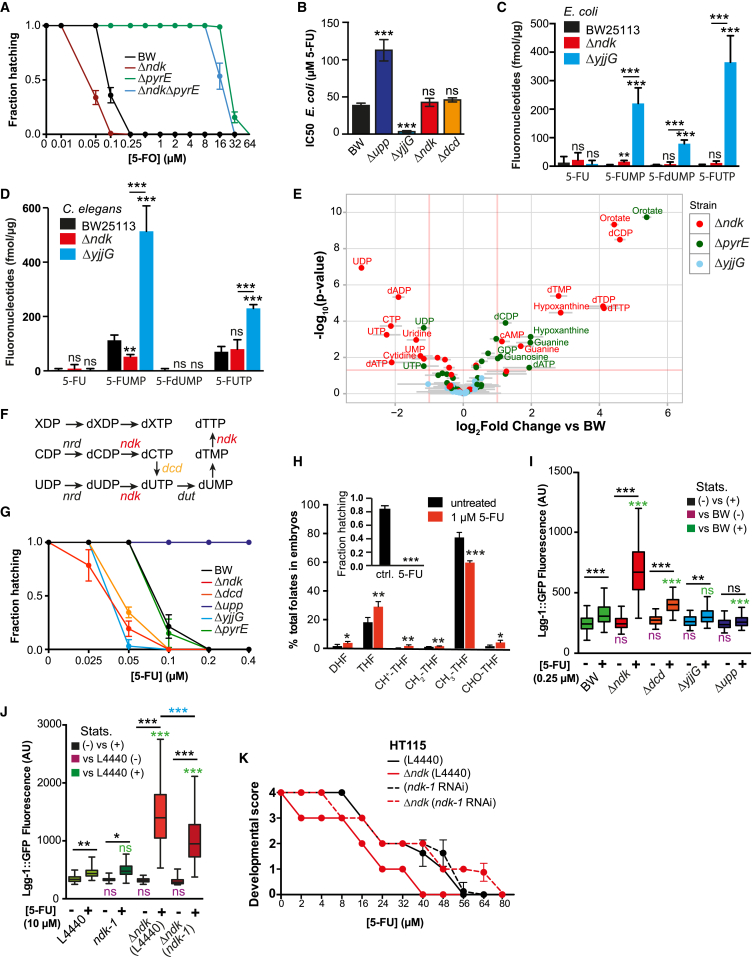
Bacteria Regulate 5-FU Effects in *C. elegans* by Two Distinct Mechanisms (A) Bacterial *ndk* effects on the efficacy of 5-FU are independent of 5-FO bioconversion mediated by *pyrE*. (B) IC_50_ values for bacterial growth with 5-FU. (C and D) LC-MS/MS quantification of fluoropyrimidines in *E. coli* (C) and *C. elegans* (D) supplemented with 50 μM 5-FU. (E) Volcano plot of nucleotide metabolism of bacterial mutants. (F) Diagram of deoxynucleotide metabolism in *E. coli*. X = G or A. (G) Bacterial deoxyribonucleotide imbalance caused by Δ*ndk* and Δ*dcd* improves 5-FU effects. (H) 5-FU alters folate metabolism homeostasis in embryos. Inset: effects of 5-FU on egg hatching of the analyzed samples. (I) Activation of autophagy (Lgg-1::GFP reporter) in embryos by 5-FU is dependent on bacteria. (J) Host *ndk-1* is required for 5-FU-induced autophagy activation by bacterial deoxynucleotide imbalance. (K) Bacterial *ndk* effects on 5-FU efficacy are mediated by the host *ndk-1* gene. Data are represented as mean ± SD. ^∗^p < 0.05; ^∗∗^p < 0.01; ^∗∗∗^p < 0.001. See also Figure S6. For statistics, see Table S6.

**Figure S6:**
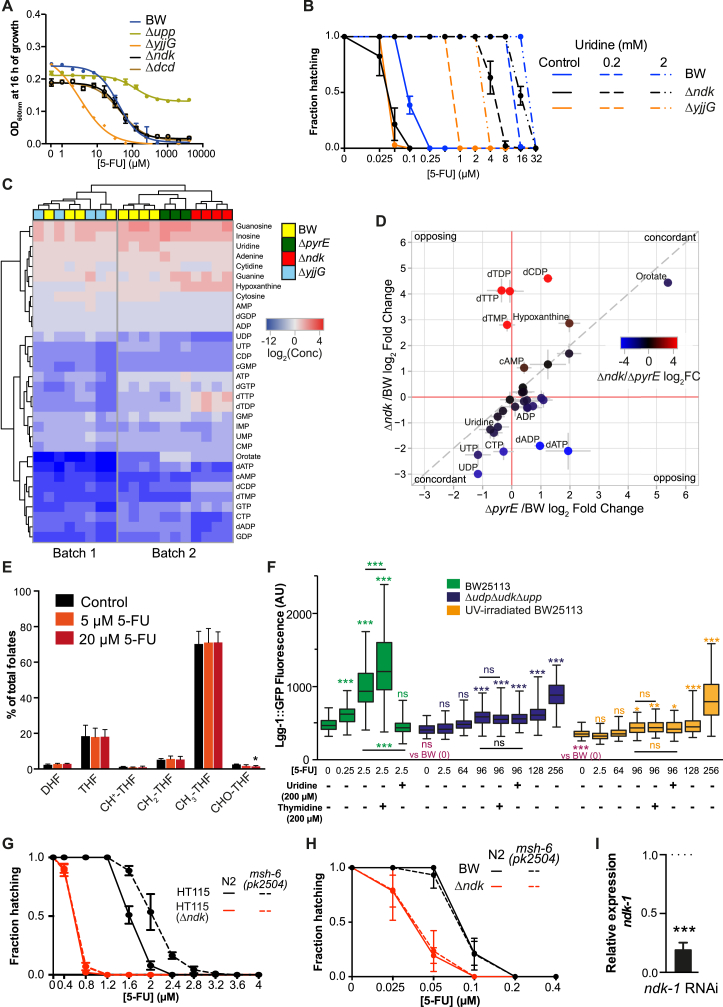
Bacterial Deoxynucleotide Metabolism Effects on 5-FU Efficacy, Related to Figure 6 (A) Effects of 5-FU on bacterial growth of sensitive Δ*yjjG*, resistant Δ*upp* and neutral Δ*ndk* or Δ*dcd.* Drug response curves were calculated using a log(inhibitor) versus response - variable slope (four parameter) model. (B) Supplementation with 200 μM and 2 mM uridine impairs 5-FU effects in worms fed Δ*yjjG and* Δ*ndk* mutants in a distinctive manner. (C) Heatmap of metabolite profiles for bacterial nucleotide metabolism mutants. Absolute metabolite levels shown by color scale, clustering done by Euclidean distance. (D) Difference in metabolite levels between Δ*ndk* (y axis) and Δ*pyrE* (x axis) mutants in comparison to BW. Color gradient shows Δ*ndk/*Δ*pyrE* logFC, indicating opposing/concordant effects of mutants on metabolite levels. Metabolites with significant changes in Δ*ndk*/BW and Δ*ndk/*Δ*pyrE* are labeled (See Table S6). (E) 5-FU does not alter folate metabolism homeostasis in whole worms at 5 and 20 μM. (F) LGG-1::GFP expression in embryos from DA2123 worms fed BW25113, UV-irradiated BW25113 and triple mutant Δ*udp*Δ*udk*Δ*upp* treated with 5-FU at various concentrations and supplemented with 200 μM uridine or thymidine. Uridine supplementation rescues 5-FU induction of autophagy while thymidine supplementation improves it in a bacterial dependent manner. (G) Increased resistance to 5-FU in the mismatch repair worm mutant *msh-6(pk2504)* is mediated by bacteria. Resistance to 5-FU by *msh-6* mutant worms is only observed when fed on HT115 but not HT115(Δ*ndk*) mutant bacteria. (H) *msh-6(pk2504)* does not confer resistance to 5-FU compared to WT worms when fed BW25113 or Δ*ndk* bacteria. (I) Efficient knockdown of *ndk-1* in worms by RNAi. Data are represented as mean ± SD. ^∗^p < 0.05; ^∗∗^p < 0.01; ^∗∗∗^p < 0.001. For statistics, see Table S6.

We found that knockout of bacterial *ndk*, the nucleoside diphosphate kinase from the deoxyribonucleotide salvage pathway, and *yjjG* from the ribonucleotide salvage pathway, improved efficacy of fluoropyrimidine treatment in worms (0.1 μM, p < 0.0001; Figures 5E, 5F, 6A, and 6G). Therefore, we probed whether *ndk* was acting in concert with the bacterial conversion of 5-FU to downstream fluoronucleotides to mediate the host response to drug. Surprisingly, knockout of *ndk* was not epistatic to *pyrE* in the mediation of 5-FO effects (Figure 6A), did not confer resistance to bacterial inhibition by 5-FU (Figures 6B and S6A), and did not alter 5-FUTP or 5-FdUMP abundance in *E. coli* or *C. elegans* (Figures 6C and 6D), the two metabolites responsible for impeding cellular division by causing RNA and DNA damage, respectively (Longley et al., 2003). In contrast, knockout of *yjjG* (1) dramatically increased fluoronucleotide abundance in both organisms (Figures 6C and 6D), possibly by impeding diversion of 5-FU metabolism to the less toxic pro-drug 5-fluorouridine (5-FUrd) (Figure 5D); (2) enhanced bacterial sensitivity to 5-FU (Figure 6B); and (3) required higher concentrations of uridine for rescuing 5-FU toxicity (Figure S6B). Altogether, these data suggest a distinct mode of action conferred by these two genes. To gain insight into the mechanism underlying such effects, we performed analysis of nucleotide metabolism by LC-MS/MS. This approach revealed discrete deoxynucleotide changes in Δ*ndk*, but not Δ*yjjG* or Δ*pyrE* (Figures 6E, S6C, and S6D). To investigate the role of microbial deoxynucleotides in modulating drug potency, we tested the function of the dCTP deaminase *dcd*, which, like *ndk*, alters dNTP pools (Figure 6F) (Maslowska et al., 2015). Knockout of *dcd*, like *ndk*, improved 5-FU efficacy on the host (Figure 6G) but did not alter the bacterial sensitivity to 5-FU (Figures 6B and S6A). Thus, bacterial deoxynucleotide imbalance mediated by *ndk* and *dcd* promotes cellular death by a distinct mechanism to increased fluoronucleotide pools in host cells (e.g., *yjjG*) (Figure 6D).

In *C. elegans*, overexpression of TS in the germline increases resistance to 5-FU effects (Kim et al., 2008). As a consequence of TS inhibition, DNA damage (Longley et al., 2003) and disruption of OCM have previously been reported for mammalian cells treated with 5-FU (Ser et al., 2016). Similarly, we observed that folate metabolism was dysregulated in embryos, but not intact worms treated with 5-FU (Figures 6H and S6E), suggesting that TS in the germline is one of the likely targets of 5-FU in *C. elegans*. DNA damage caused by cancer drugs induces autophagy-mediated cell death in human cells (Zeng and Kinsella, 2011). Similarly, DNA damage checkpoint activation by 5-FU in *C. elegans* induces autophagy, measured by changes in *lgg-1*::GFP fluorescence, the worm ortholog of atg8/LC3 (SenGupta et al., 2013). Therefore, we investigated the role of bacteria in the activation of autophagy by 5-FU and found that autophagy was activated by 5-FU in a dose- and bacterial-dependent manner (Figure S6F). Also, Δ*ndk* and Δ*dcd* but not Δ*yjjG* synergized the effects of 5-FU to induce autophagy (Figure 6I) despite similar effects on embryonic lethality (Figure 6G), further supporting a distinct mechanism to induce cell death in host cells. To probe the involvement of the DNA damage mismatch repair (MMR) pathway in the activation of autophagy (SenGupta et al., 2013), we tested the MMR-deficient mutant *msh-6(pk2504).* We found that *msh-6* improved resistance to 5-FU, as previously reported (SenGupta et al., 2013), but this effect was dependent on bacterial metabolism, in particular, deoxynucleotide metabolism. *msh-6* mutants were only resistant to 5-FU when fed HT115 (Figure S6G), but not BW25113 (Figure S6H) or Δ*ndk* (Figures S6G and S6H). Thus, imbalanced bacterial deoxynucleotide pools improve 5-FU efficacy in the host independently of the MMR. So, how is autophagy activated by bacterial deoxynucleotide pools in the context of 5-FU treatment? Given the role of *ndk* in regulating deoxynucleotide metabolism, we tested whether the worm ortholog *ndk-1* plays a role in mediating these effects. Efficient knockdown of *ndk-1* by RNAi (Figure S6I) reduced fertility (data not shown) but did not contribute to 5-FU effects on the host (Figures 6J and 6K). *ndk-1* RNAi solely decreased the activation of autophagy (Figure 6J) and abolished the improvement in 5-FU efficacy conferred by knockout of bacterial *ndk* (Figure 6K). Thus, host *ndk-1* regulates effects of microbial deoxynucleotides to modulate drug potency. This implies an alternative role for NDK rather than its direct involvement in the metabolism of fluoropyrimidines. Overall, we show that bacteria mediate two modes of cellular death in the host either by an increase of RNA-damaging FUTP (e.g., Δ*yjjG)* or by sensitizing the DNA-damaging effects of 5-FU (e.g., Δ*ndk*, Δ*dcd*).

### Anti-hyperproliferation and Pro-survival Effects of 5-FU Are Dependent on Bacteria

**Figure 7:**
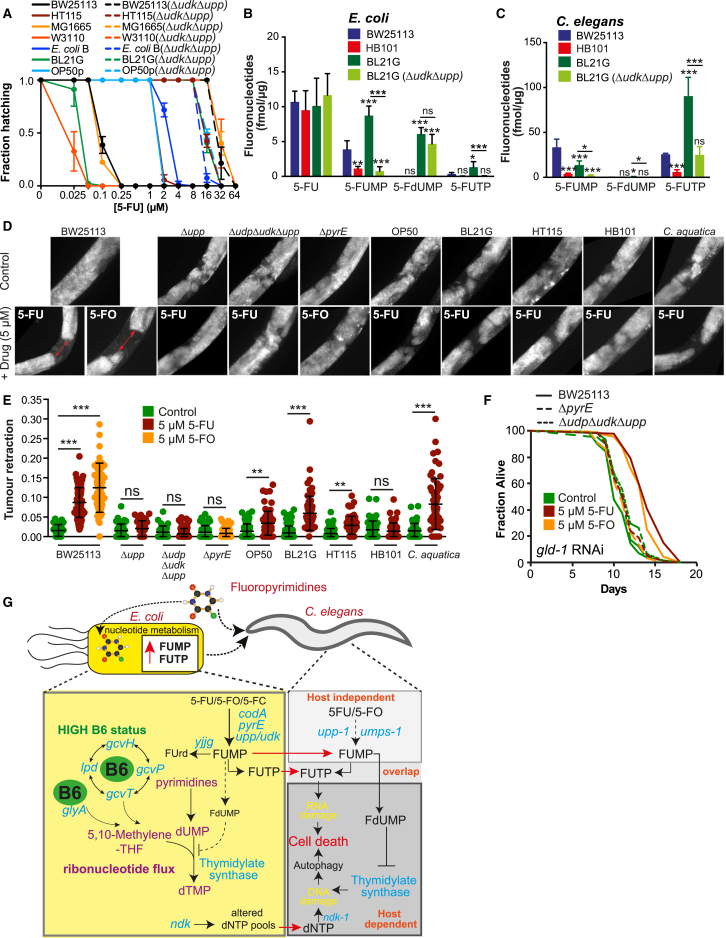
5-FU Improves Survival and Reduces Germline Hyper-Proliferation of *gld-1* RNAi Worms (A) *upp* and *udk* mediate the effects of 5-FU regardless of bacterial genetic background. (B and C) LC-MS/MS quantification of fluoropyrimidines in *E. coli* (B) and *C. elegans* (C) supplemented with 50 μM 5-FU. (D) Representative images of DAPI-stained hyperproliferative gonads of *gld-1* RNAi worms. Images were rotated and aligned for ease of comparison. (E) 5-FU and 5-FO reduce tumor size in a bacterial-dependent manner. Tumor retraction, distance between gonad arms at midsection/distance between gonad loops. (F) 5-FU and 5-FO extends the lifespan of *gld-1* RNAi worms when fed on control bacteria but not ribonucleotide mutants. (G) Diagram summarizing the effects of 5-FU on the *C. elegans*/*E. coli* holobiont. Data are represented as mean ± SD. ^∗^p < 0.05; ^∗∗^p < 0.01; ^∗∗∗^p < 0.001. See also Figure S7. For statistics, see Table S7.

**Figure S7:**
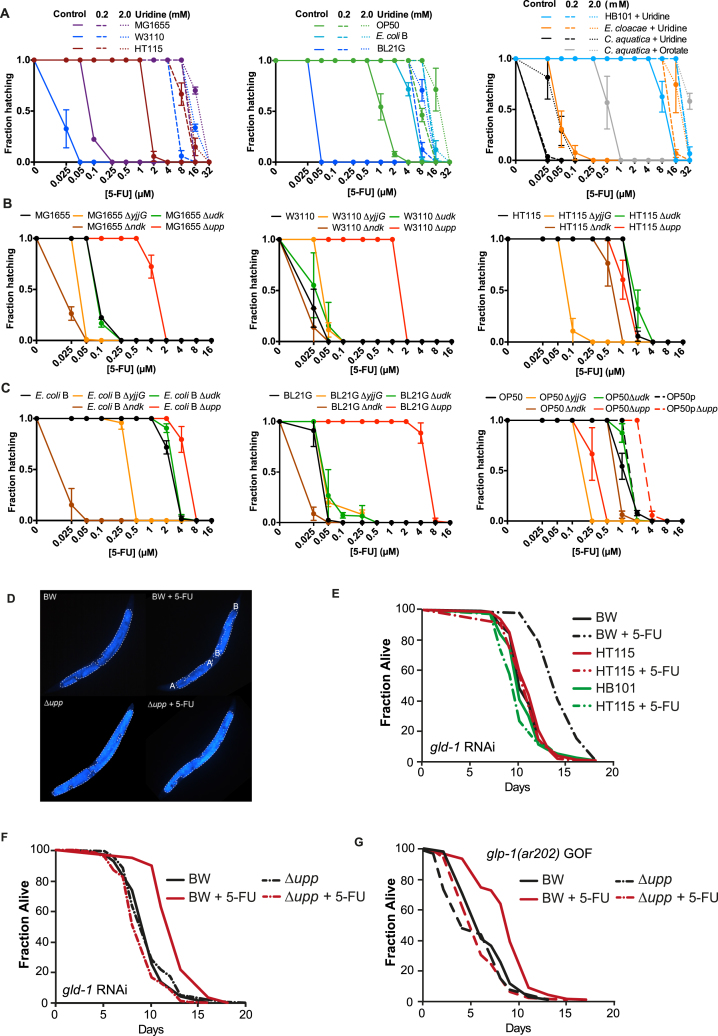
5-FU Effects on Tumor Size and Survival of the Host Are Dependent on Bacterial Genotype, Related to Figure 7 (A) Supplementation with uridine and orotate (200 μM or 2 mM) impairs 5-FU effects in worms fed diverse bacterial strains. (B) Role of bacterial *yjjG*, *udk*, *upp*, and *ndk* in *E. coli* K-12 strains in the regulation of 5-FU effects on the host. (C) Role of bacterial *yjjG*, *udk*, *upp*, and *ndk* in *E. coli* B strains in the regulation of 5-FU effects on the host. OP50p refers to OP50 prototroph for uracil/uridine. (D) Representative 10x images of DAPI stained *gld-1* RNAi whole worms treated for 4 days with 5-FU and fed control or Δ*upp* mutant bacteria. Tumor retraction as measured by distance (A’ to B’)/(A to B). (E) 5-FU extends the lifespan of *gld-1* worms when fed on BW25113 bacteria but not HB101 or HT115. (F and G) 5-FU extends the lifespan of *gld-1* (F) and *glp-1* (G) gain-of-function worms when fed on control bacteria, but not Δ*upp* mutants. Data are represented as mean ± SD. For statistics, see Table S7.

Does ribonucleotide metabolism of fluoronucleotides account for differences observed between bacterial strain serotype (Figures 1A and 1B)? We find that Δ*upp*Δ*udk* double knockouts, which fully abolish salvage ribonucleotide metabolism, confer similar worm MICs for 5-FU regardless of bacterial genetic background (Figure 7A), and the supplementation with uridine or orotate rescues 5-FU effects in all strains tested (Figure S7A). Also, measurements of metabolized fluoronucleotides both in bacteria and *C. elegans* correlate with drug efficacy on the host (Figures 7B and 7C). This implies that bacterial ribonucleotide metabolism is the bottleneck of 5-FU action and other pathways modulate its flux. Interestingly, and contrarily to the knockout of bacterial *ndk* that always increases 5-FU efficacy on the host, we found that single mutations of *yjjG*, *udk*, and *upp* have strain-dependent effects on 5-FU toxicity (Figures S7B and S7C). For example, knockout of *upp* on OP50 or an OP50-uracil prototroph, with restored de novo pyrimidine biosynthesis, has opposite effects on 5-FU efficacy in the host suggesting a complex interplay between bacterial salvage and de novo ribonucleotide metabolism in regulating host toxicity (Figure S7C).

Next, we tested the anti-proliferative effects of 5-FU on tumor-like germlines in the worm. Knockdown of *gld-1* or gain of function of *glp-1* in *C. elegans* impairs oocyte development, resulting in a hyperproliferative germline and ultimately death (Pinkston et al., 2006). Consistent with our previous findings, 5-FU or 5-FO effects on tumor size were dependent on bacterial genotype (Figures 7D, 7E, and S7D). 5-FU and 5-FO also extended *gld-1* worm lifespan when fed on BW25113 bacteria (+34.4%, p < 0.001; Figure 7F, Table S7) but importantly not with Δ*udp*Δ*udk*Δ*upp or* Δ*pyrE* mutants, respectively (–2.27%, p = 0.2830; −0.79%, p = 0.9341). Also, 5-FU did not extend survival of worms fed HT115 and HB101 (Figure S7E). Similar effects were obtained for *gld-1* and the gain-of-function *glp-1* worms fed Δ*upp E. coli* (Figures S7F and S7G). These results highlight that the pro-survival and anti-proliferative properties of fluoropyrimidines depend on bacterial metabolism, a finding of potential clinical relevance to humans.

## Discussion

In this study, we have demonstrated how microbes and dietary cues modulate the efficacy of fluoropyrimidines on the *C. elegans* host.

### Chemical-Holobiont Screens for Drug Effects on Host Physiology

Historically, pharmacology has solely considered host biology when developing new therapeutics, which may contribute to the high degree of failure observed for drugs undergoing clinical trial. While it is accepted that gut microbiota are capable of altering drug pharmacodynamics (Spanogiannopoulos et al., 2016), probing the complex relationships between host, microbe, and drug has proved difficult. Here, we used a nematode-microbe model to unravel the contribution of bacterial genes in host responses to anti-cancer drugs by testing a combination of bacterial genetics, dietary sources, and chemical compounds. The advantages of the metabolically and evolutionarily conserved *C. elegans* model are clear: the convenience of sustaining animals on defined bacterial populations, together with the ease of genetic manipulations and biochemical analysis in both organisms, allows rigorous investigation of host-microbe interactions by rapid screening that is not possible in higher models. This system may be of particular use for pre-clinical screening of drug interactions in the context of host and microbe, a task usually studied in vitro or by analyzing candidate compounds using in vitro fecal models (Spanogiannopoulos et al., 2016), or for designing bacteria for drug delivery, a prospect for future personalized medicine. While our screening approach of incorporating the host as a biological readout validated findings obtained by more traditional microbial high-throughput genetic and chemical genetic approaches, we provide striking insights into host-microbe interactions that could not be uncovered by studying microbes in isolation.

Another advantage of our approach is the evolutionarily conserved effects of 5-FU on host metabolism, at similar physiological drug concentrations to that observed in human plasma after treatment or at concentrations capable of inhibiting TS in colon cancer cells (Ser et al., 2016). In fact, similar metabolic changes were observed in the serum of tumorous mice treated with 5-FU (Ser et al., 2016) and worms treated with 5-FU. Changes in metabolites from pyrimidine (e.g., uracil, orotate), one-carbon (e.g., methionine, homocysteine, L-serine), and carnitine metabolism (e.g., L-acetylcarnitine, isovalerylcarnitine) were observed (Figures S1K and S1L). Most importantly, the model proved valuable in identifying key biological processes at the host-microbe interface (bioconversion and host-microbe metabolic complementation for drugs), bacterial pathways responsible for drug efficacy (e.g., ribonucleotide (Garcia-Gonzalez et al., 2017 [this issue of *Cell*]), vitamin B_6_, and OCM), and dietary cues affecting drug metabolism (e.g., pyrimidines) (Figure 7E).

### Role of the Microbiota in Modulating Fluoropyrimidine Efficacy

We report that bacterial activation of fluoropyrimidines by direct metabolic conversion is critical for optimal drug efficacy in worms. Given that the microbiota is a key determinant of host health and a source of inter-individual variability (Nicholson et al., 2012), and given the highly conserved nature of nucleotide metabolism across bacterial taxa (Peregrín-Alvarez et al., 2009), our results raise the possibility that microbes might similarly influence the action of 5-FU and predict cancer therapy outcomes in patients. In recent years, there have been several reports of microbial influence on drug efficacy (reviewed by Spanogiannopoulos et al., 2016), some in the context of cancer therapy. For example, gut microbes have been reported to reactivate the cancer drug irinotecan, causing toxicity. Also, gut microbes isolated from human feces are capable of converting 5-FC to 5-FU, but whether this phenomenon contributes to drug efficacy in the host has never been tested until now. Similarly, we demonstrate that *E. coli* can convert not only 5-FC, but also other clinically relevant fluoropyrimidine prodrugs such as 5-FU and capecitabine. Importantly, worm responses to fluoropyrimidines were strongly contingent on their microbes, with pharmacodynamics varying by as much as 40-fold on different bacterial strains and disruption of bacterial metabolism leading to changes as great as 256-fold. We have revealed that inhibition of bacterial ribonucleotide metabolism drastically antagonizes drug efficacy, while inhibition of deoxyribonucleotide metabolism improves it, an effect also regulated by dietary pyrimidines. Similar mechanisms have been observed in mammals, where dietary arginine regulates the inactivation of the cardiac drug digoxin by the gut microbe *E. lenta* (Haiser et al., 2013).

Here, we have discovered that the *C. elegans*-*E. coli* holobiont acts together as a single unit in the context of drug metabolism, showing biochemical complementation for the mediation of fluoropyrimidine effects on host physiology (Figure 7G).

### Role of Diet and Drugs in Fluoropyrimidine Efficacy

Interventions that disrupt the microbiota, such as antibiotics, impair the response of tumors to CpG-oligonucleotide immunotherapy and platinum chemotherapy (Iida et al., 2013). Additionally, unforeseen interactions between the antiviral drug sorivudine and 5-FU prodrugs have led to patient deaths due to microbiome-drug interactions (Okuda et al., 1997). Consistent with these findings, our data show that the anti-diabetic drug metformin, which possesses anti-cancer properties, inhibits bacterial OCM, thereby reducing 5-FU efficacy in *C. elegans*. Given the interaction of metformin with the human microbiota (Cabreiro, 2016), these results raise the possibility that co-therapies for cancer might yield undesired outcomes if host-microbe-drug interactions are not taken into account.

In addition to drugs, we observed that dietary nutrients (e.g., pyrimidines, vitamin B_6_) can alter the efficacy of 5-FU. Vitamins produced by gut microbes (e.g., vitamin B_12_) are a determining factor in shaping microbial communities (Degnan et al., 2014). Likewise, our data show that in bacteria, B_6_ regulates ribonucleotide flux via modulation of OCM.

### Bacteria as Regulators of 5-FU-Induced Cell Death: A Double-Edged Sword

The magnitude and balance of deoxynucleotide pools are important for replication fidelity (Maslowska et al., 2015). Here, we show that alterations in deoxynucleotide pools caused by mutations in *dcd* and *ndk* do not sensitize bacterial mutants to the DNA damage agent 5-FU but increase efficacy of treatment in the host. Our results illustrate that the holobiont metabolic complementation occurs at two levels in the context of drugs: (1) in their bioconversion and by regulating the availability of RNA-damaging fluoronucleotides such as FUTP to the host, and (2) in the supply of regulatory metabolites that synergize with 5-FU-induced DNA damage. Consistent with our observations, it was previously reported that bacterial nucleotide metabolism regulates *C. elegans* germline proliferation (Chi et al., 2016).

Cancer treatments can activate either pro-survival or pro-death autophagy activity in tumor cells, an effect dependent on the cellular context (Zeng and Kinsella, 2011), but this mechanism is not fully understood. Our data suggest a divergent role for dNTP pool imbalance between prokaryotes and eukaryotes and a mechanism where deoxynucleotide pools from non-self to self can amplify autophagy-mediated cell death inflicted by fluoropyrimidines on eukaryotic cells. In contrast, increased dNTP pools desensitize human cancer cells to rapamycin-induced autophagy (Chen et al., 2014). Altogether, these findings implicate dNTP pools in the regulation of autophagy by drugs, a mechanism of relevance to cancer biology and human health.

Our chemical-genomic-holobiont screening approaches highlight that bacteria play key roles in the efficacy of a drug in an animal model and illustrate a critical missing component in our understanding of the mechanistic basis of drugs in the treatment of disease. In humans, the identification of gut microbiota members responsible for these mechanisms and their regulation by dietary supplements could have a dramatic impact on treatment outcome.

## STAR★Methods

### Key Resources Table

**Table:** 

REAGENT or RESOURCE	SOURCE	IDENTIFIER
**Bacterial and Virus Strains**		

*E. coli:* W3110: F-, λ-, rph-1, IN(rrnD, rrnE)	CGSC	CGSC: 4474
W3110 Δ*yjjG::kan*	This Study	N/A
W3110 Δ*upp::kan*	This Study	N/A
W3110 Δ*udk::kan*	This Study	N/A
W3110 Δ*ndk::kan*	This Study	N/A
W3110 Δ*upp* Δ*udk::kan*	This Study	N/A
*E. coli*: MG1655: F-, λ-, rph-1, ilvG-, rfb-50	CGSC	CGSC: 6300
MG1655 Δ*yjjG::kan*	This Study	N/A
MG1655 Δ*upp::kan*	This Study	N/A
MG1655 Δ*udk::kan*	This Study	N/A
MG1655 Δ*ndk::kan*	This Study	N/A
MG1655 Δ*upp* Δ*udk::kan*	This Study	N/A
*E. coli*: HT115: mcrA, mcrB, IN(rrnD, rrnE)1, rnc14::Tn10 λ(DE3)	CGC	CGC: 8854; RRID:WB-STRAIN:HT115(DE3)
HT115 Δ*ndk::kan*	This Study	N/A
HT115 Δ*yjjG::kan*	This Study	N/A
HT115 Δ*upp::kan*	This Study	N/A
HT115 Δ*udk::kan*	This Study	N/A
HT115 Δ*upp* Δ*udk:kan*	This Study	N/A
*E. coli*: HB101: supE44 hsdS20(rB-mB-) recA13 ara-14 proA2 lacY1 galK2 rpsL20 xyl-5 mtl-1	CGSC	CGSC: 12554
*E. coli*: BW25113: F-, Δ(araD-araB)567, ΔlacZ4787(::rrnB-3), λ-, rph-1, Δ(rhaD-rhaB)568, hsdR514	CGSC	CGSC: 7636
Keio collection: Single-gene knockout mutants in *E. coli* BW25113 background	NBRP	https://shigen.nig.ac.jp/ecoli/strain/resource/keioCollection/list/
BW25113 Δ*metE* Δ*metH::kan*	This Study	N/A
BW25113 Δ*pdxJ* Δ*gcvP::kan*	This Study	N/A
BW25113 Δ*pdxJ* Δ*upp::kan*	This Study	N/A
BW25113 Δ*udk* Δ*udp::kan*	This Study	N/A
BW25113 Δ*pdxK* Δ*pdxJ::kan*	This Study	N/A
BW25113 Δ*upp* Δ*udp::kan*	This Study	N/A
BW25113 Δ*upp* Δ*udk::kan*	This Study	N/A
BW25113 Δ*upp* Δ*tdk::kan*	This Study	N/A
BW25113 Δ*ndk* Δ*pyrE::kan*	This Study	N/A
BW25113 Δ*nupG* Δ*nupC::kan*	This Study	N/A
BW25113 Δ*udk* Δ*upp* Δ*gcvP::kan*	This Study	N/A
BW25113 Δ*udk* Δ*upp* Δ*pdxJ::kan*	This Study	N/A
BW25113 Δ*udp* Δ*upp* Δ*udk::kan*	This Study	N/A
BW25113 Δ*udk* Δ*upp* Δ*tdk::kan*	This Study	N/A
*E. coli*: OP50: Uracil auxotroph	CGC	CGC: 11077; RRID:WB-STRAIN:OP50
OP50 Δ*yjjG::kan*	This Study	N/A
OP50 Δ*upp::kan*	This Study	N/A
OP50 Δ*udk::kan*	This Study	N/A
OP50 Δ*ndk::kan*	This Study	N/A
OP50p (uracil prototroph)	This Study	N/A
OP50p (uracil prototroph) Δ*upp::kan*	This Study	N/A
OP50p (uracil prototroph) Δ*upp* Δ*udk::kan*	This Study	N/A
OP50-MR (metformin resistant)	(Cabreiro et al., 2013)	N/A
*E. coli:* B Wildtype	CGSC	CGSC: 2507
*E. coli*: B Δ*yjjG::kan*	This Study	N/A
*E. coli*: B Δ*upp::kan*	This Study	N/A
*E. coli*: B Δ*udk::kan*	This Study	N/A
*E. coli*: B Δ*ndk::kan*	This Study	N/A
*E. coli*: B Δ*upp* Δ*udk::kan*	This Study	N/A
*E. coli*: BL21G: F ompT hsdS(rB mB) *dcm*+ Tetr *gal* λ(DE3) *endA Hte*	Fisher Scientific	Cat# 50-125-348
BL21G Δ*upp* Δ*udk::kan*	This Study	N/A
BL21G Δ*upp Δudk* Δ*udp::kan*	This Study	N/A
BL21G Δ*yjjG::kan*	This Study	N/A
BL21G Δ*upp::kan*	This Study	N/A
BL21G Δ*ndk::kan*	This Study	N/A
BL21G Δ*udk::kan*	This Study	N/A
*Comamonas aquatica*: DA1877	CGC	CGC: 7905; RRID:WB-STRAIN:DA1877
*Enterobacter cloacae*: LMG 2783	BCCM	ATCC 13047

**Chemicals, Peptides, and Recombinant Proteins**		

5-Fluorouracil ≥ 99%	Sigma-Aldrich	Cat# F6627
5-Fluoro-2′-deoxyuridine	Alfa Aesar	Cat# L1497
5-Fluorocytosine ≥ 99%	Acros Organics	Cat# 258340010
5-Fluoroorotic acid 98%	ThermoFisher Scientific	Cat# R0811
Capecitabine ≥ 99%	Santa Cruz Biotechnology	Cat# SC-205618
Metformin (1,1 Dimethylbiguanide hydrochloride)	Sigma-Aldrich	Cat# D150959
D3 pyridoxal phosphate	Buchem BV	N/A
D3 pyridoxal ≥ 98%	Sigma-Aldrich	Cat# 705187
D2 pyridoxine > 98%	CDN Isotopes	Cat# D-6819
D3 Pyridoxamine > 98%	Sigma-Aldrich	Cat# 705322
Cytidine-13C9,15N3 5′triphosphate	Sigma-Aldrich	Cat# 645699
Uridine-13C9,15N2 5′-triphosphate	Santa Cruz Biotechnology	Cat# SC-301963A

**Critical Commercial Assays**		

Pierce™ BCA Portein Assay Kit	ThermoFisher Scientific	Cat# 23250
Qubit® RNA HS Assay Kit	ThermoFisher Scientific	Cat# Q32852
Qubit® Protein Assay Kit	ThermoFisher Scientific	Cat# Q33211
GenElute Plasmid Miniprep Kit	Sigma-Aldrich	Cat# PLN70
Direct-zol RNA MiniPrep	Zymo Research	Cat# R2052
iScript Reverse Transcription Supermix for RT-qPCR	Bio-Rad	Cat# 1708841
PrecisionPLUS 2X qPCR Mastermix	PrimerDesign	Cat# PrecisionPLUS-R-SY
Phusion High-Fidelity DNA Polymerase	ThermoFisher Scientific	Cat# F530S

**Experimental Models: Organisms/Strains**		

*C. elegans*: N2 Bristol	CGC	CGC: 10570
*C. elegans*: GH636: *umps-1(zu456)III*	CGC	CGC: 19419; RRID:WB-STRAIN:GH636
*C. elegans*: GC833: *glp-1(ar202)III*	CGC	CGC: 15458; RRID:WB-STRAIN:GC833
*C. elegans*: NL2099: *rrf-3(pk1426)II*	CGC	CGC: 10766; RRID:WB-STRAIN:NL2099
*C. elegans*: NL2511: *msh-6(pk2504)*	CGC	CGC: 10771; RRID:WB-STRAIN:NL2511
*C. elegans*: DA2123: adIS2122[*lgg-1p::GFP::lgg-1* + *rol-6 (su1006*)]	CGC	CGC: 15849; RRID:WB-STRAIN:DA2123

**Oligonucleotides**		

For information regarding oligonucleotide sequences used in this study, please refer to Table S1*.*	This Study	Table S1; http://dx.doi.org/10.1016/j.cell.2017.03.040

**Recombinant DNA**		

FLP recombinase, temp-sensitive replication: pCP20	CGSC	CGSC: 7629
Cloning expression vector, medium copy no.: pACYC184	CGSC	CGSC: 12139
BW25113 upp cloned into pACYC184: pUpp	This Study	N/A
Ahringer *C. elegans* RNAi library: RNAi control plasmid: pL4440	Source BioScience	http://www.sourcebioscience.com/products/life-science-research/clones/rnai-resources/c-elegans-rnai-collection-ahringer/
Ahringer *C. elegans* RNAi library: RNAi ndk-1 (F25H2.5) knockdown: pL4440-ndk-1	Source BioScience	http://www.sourcebioscience.com/products/life-science-research/clones/rnai-resources/c-elegans-rnai-collection-ahringer/
Vidal *C. elegans* RNAi library: RNAi gld-1 (T23G11.3) knockdown: pL4440-gld-1	Source BioScience	http://www.sourcebioscience.com/products/life-science-research/clones/rnai-resources/c-elegans-orf-rnai-resource-vidal/
Alon GFP transcriptional reporter library: ndk promoter GFP reporter: pNdk::GFP	Dharmacon	Cat# PEC3876
Alon *E. coli* GFP transcriptional reporter library: udk promoter GFP reporter: pUdk::GFP	Dharmacon	Cat# PEC3876
Alon *E. coli* GFP transcriptional reporter library: upp promoter GFP reporter: pUpp::GFP	Dharmacon	Cat# PEC3876

**Software and Algorithms**		

R (v3.2.3)	R consortium	https://www.r-project.org
GraphPad Prism 6	GraphPad Software, Inc.	https://www.graphpad.com/scientific-software/prism/
JMP 11	SAS Institute Inc.	http://www.jmp.com/en_be/software/data-analysis-software.html

### Contact for Reagent and Resource Sharing

Further information and requests for reagents may be directed to, and will be fulfilled by the Lead Contact Filipe Cabreiro (f.cabreiro@ucl.ac.uk).

### Experimental Model and Subject Details

#### Nematode and Bacterial Strains

*C. elegans* strains N2 Bristol, GH636 *umps-1(zu456)III*, GC833 *glp-1(ar202)III*, NL2099 *rrf-3(pk1426)II*, NL2511 *msh-6(pk2504)* and DA2123 adIS2122[*lgg-1p::GFP::lgg-1* + *rol-6 (su1006)*] were obtained from the Caenorhabditis Genetics Center (CGC). The *E. coli* Keio Knockout Collection (Baba et al., 2006) (odd numbered strains) was obtained from the National BioResource Project. Other bacterial strains obtained from the Coli Genetic Stock Center, and bacterial strains created for this study are listed in Table S1. Both bacterial and nematode mutants were re-confirmed by PCR or snPCR using primers listed in Table S1.

#### Nematode Culture Conditions

Worms were maintained and raised at 20°C, unless otherwise stated, on nematode growth medium (NGM). Where indicated molten agar was supplemented with fluoropyrimidines (0.01, 0.05, 0.1, 0.25, 0.5, 1, 2, 4, 8, 16, 32, 64 μM), nucleotides (200 μM, 2 mM) and B_6_ vitamers (10 μM, 1 mM). Maintenance of the GH636 strain was achieved on NGM plates supplemented with 100 mM uridine. For UV and heat treatment of bacteria, an overnight culture of the indicated bacterial strain grown at 37°C was centrifuged at 5000 rpm for 30 min at 4°C, then resuspended in phosphate buffered saline (PBS) at a final OD_600nm_ = 24. For UV treatment, bacteria were irradiated for 30 min on a CL-1000 Ultraviolet Crosslinker (UVP) containing bulbs irradiating at 254 nm; for heat treatment, bacteria were incubated for 30 min at 70°C in a water bath. For RNAi experiments, worms were kept for two generations on NGM plates containing 1 mM IPTG (isopropyl β-d-1-thiogalactopyranoside) seeded with *E. coli* HT115(DE3) or HT115(DE3) Δ*ndk* expressing RNAi constructs in the pL4440 feeding vector. RNAi clones were verified by sequencing and efficacy of knockdown was determined by qRT-PCR (Figures S3D and S6I).

#### Bacterial Strain Construction

Bacterial strains used and generated in this study are shown in the Key Resources Table. *E. coli* single deletion mutants were obtained from the Keio collection (Baba et al., 2006). Strains with multiple mutations were constructed by removing the kanamycin resistant marker from single Keio clones by transformation with pCP20 and subsequent transfer of kanamycin resistant-tagged mutations via P1*vir* phage-mediated transduction. All bacterial mutants were confirmed by colony PCR using the primers detailed in Table S1. In general, binding sites of the -cseq-F and -R primers are located up- and down-stream of the mutation site, respectively, and were used to confirm kanamycin-sensitive mutants, while the K1, which binds to the kanamycin resistance gene, was used in conjunction with the appropriate -cseq-F primer to confirm kanamycin-resistant mutants.

For the generation of BW25113-GFP strains we used bacterial clones containing promoter-fused plasmids from the library of transcriptional fusions of gfp from the Uri Alon lab. The MG1655 strains containing promoter-fused plasmids for *udk, ndk*, and *upp* genes (see Key Resources Table plasmid list) were selected from the library and each plasmid was extracted using a GenElute Plasmid Miniprep Kit (Sigma Aldrich). The resulting plasmid DNA was confirmed by PCR, using the -cseq-F primer and the Primer 1 from pUA66 as a reverse primer (Table S1). Following confirmation, each plasmid was transformed into the BW25113 strain.

For the generation of pUpp complementation plasmid, the DNA sequence of the *upp* gene, including its promoter and terminator, was amplified from the BW25113 chromosome using Phusion High Fidelity DNA Polymerase (Thermo Fisher Scientific) and the primers listed in Table S1. The DNA fragment was cloned into pACYC184 at the HindIII and BamHI sites, the inserted sequence confirmed by PCR using primers pACYC -Hind-F and -Bam-R.

### Method Details

#### Lifespan Analysis

Axenic worm eggs were obtained by treating healthy gravid WT adults with alkaline hypochlorite. Bleached eggs were allowed to develop until the L4 larval stage on HT115(DE3) L4440 or *gld-1* RNAi *E. coli* at 25°C. Efficacy of *gld-1* knockdown was confirmed by tumorigenicity of the gonads and by the absence of embryos/oocytes. Animals (*gld-1* RNAi or *glp-1*(gof)) were transferred to control and 5-FU treated plates seeded with BW25113 or BW25113 Δ*upp* and maintained at 25°C for the whole duration of the lifespan. Worms were transferred to fresh plates every 4 days. Animals that did not display tumorous gonads were removed from the trial at day 1 of adulthood and the ones that showed protruded vulvas were censored. Survival was monitored every 1-2 days and worms were scored as dead if they did not respond to prodding with a platinum wire. Statistical analysis was performed by the log-rank test using JMP 11 software (SAS Institute).

#### Hatching Assays

Assays were performed in 12-well plates. *C. elegans* were raised from L1 stage on control (BW25113), other WT or mutant *E. coli* strains for 2 days at 20°C. Two L4-stage worms were transferred to each well containing seeded NGM agar supplemented with varying concentrations of 5-FU or other drugs. Adult worms were removed after a 24 hr incubation at 25°C. After another 24 hr incubation period, the hatching ratio was scored as the fraction (%) of developed larvae from the initially laid eggs, and presented as the mean ± SD from at least 3 independent biological replicates with at least 100 animals per data point. Statistical analysis was performed by group analysis one per row multiple t tests using Graphpad Prism 6 software.

#### Developmental assay

NGM agar plates containing the appropriate drug concentration were prepared, seeded, and incubated as described for the Host-Microbe-Drug Screen. Visual scoring of nematode development was based on a 5 level system, as follows:

0 = Complete ablation of development (i.e., arrested at L1 stage),
1 = L2/L3-sized
2 = L4/young adult-sized
3 = Fertile adults, no progeny
4 = Complete development, fertile adults with progeny (comparable to no drug control)

#### *C. elegans* bacterial-consumption assays

Bacteria grown overnight in LB-Miller broth were washed twice in NGM broth, normalized to OD_595nm_ = 3.1 and added to 96-well microtiter plates (100 μL/well, OD_595nm_∼0.6). Antibiotics (Carbenicillin, 100 μg/mL; Streptomycin, 100 μg/mL; Penicillin, 100 μg/mL) were included to inhibit bacterial growth. Synchronized L1 nematodes were added at 800-1000 animals per well. Plates were incubated at 25°C, 400 rpm and the absorbance at OD_595nm_ was measured at 16, 20, 24 and 28 hr using a Tecan Infinite Pro M200 microplate reader and Magellan v7.2 software. Rates of consumption were expressed as the decrease in OD_595nm_ units per hour.

#### *C. elegans* Fluorescence Microscopy

Quantitation of 5-FU growth inhibition was achieved by using the constitutive and robust expression of the GFP::LGG-1 throughout all worm developmental stages and after freezing as a readout of worm size. L1 larvae were placed in 96-well plates containing 5-FU supplemented NGM and seeded with BW25113, BW25113 Δ*upp*, and the respective complemented strains. After 48 hr incubation at 20°C, worms from each well were resuspended in PBS, transferred to a 96-well plate and frozen before imaging. GFP intensity as a measure of area was quantified as the pixel density in the entire cross sectional area of each worm from which the background pixel density was subtracted. 30 worms per condition in 3 biological replicates were imaged.

Alternatively, L1s were raised to the L4-stage on the appropriate bacterial strains, then transferred to 12-well plates containing 5-FU and respective controls to lay eggs for 24 hr at 25°C (similarly to the hatching assays). The levels of autophagy (GFP::LGG-1) in embryos were measured based on whole egg GFP intensity. At least 40 embryos per condition in 3 biological replicates were imaged.

For the effects of 5-FU on tumorous gonad size, day-4 adult *gld-1* RNAi animals for each experimental drug and bacterial condition were fixed in 100% methanol for 5 min on ice. Samples were stained with 500 mg/mL DAPI for 30 min in the dark, followed by 3x M9 buffer washes, and placed on 2% agarose pads before imaging. Images were taken of the entire animal using the 10x objective. Tumor retraction was measured as the distance between the two arms of the gonad at the midpoint of the cross sectional area normalized to the distance between the loops of the gonads. At least 45 worms per condition in 3 biological replicates were imaged.

All images were taken using a Zeiss Axioskop 2 Plus microscope with a DAPI filter cube (excitation: 358 nm; emission: 463 nm) or a GFP filter cube (excitation: 470 nm; emission: 525 nm), an Hamamatsu ORCA-ER digital camera, and further analyzed using Volocity 6.3 software (PerkinElmer). Statistical analysis was performed by one-way ANOVA using GraphPad Prism 6 software.

#### Quantitative RT-PCR

Transcriptional measurements of *C. elegans* were performed in synchronized L4 hermaphrodites fed on diverse bacterial strains and conditions. For total RNA extraction, worms were disrupted in TRI Reagent (Zymo Research) with lysing matrix D (MP Biomedicals), and flash-frozen in liquid nitrogen, then stored at –80°C overnight. After a thaw-freeze-thaw procedure, samples were homogenized using ThermoMixer C (Eppendorf) at 2000 rpm for 10 min at 4°C. Total RNA were extracted using Direct*-*zol MiniPrep (Zymo Research) and purified of DNA by in-column DNase I treatment. RNA quantification was achieved using Qubit*®* RNA HS Assay Kit (Thermo Fisher Scientific). cDNA synthesis was performed using iScript Reverse Transcription Supermix (Bio-Rad). Quantitative PCR was performed with PrecisionPLUS 2x qPCR MasterMix (PrimerDesign) using a LightCycler® 480 Real-Time PCR system (Roche). Primer sequences for each gene (Table S1) were previously optimized over a linear range of cDNA concentrations. Relative transcriptional abundance of target genes was calculated using the ΔCt method and was normalized to averaged mRNA levels of the housekeeping gene *cdc-42*. At least 3 independent biological replicates for each condition were measured per gene. Statistical significance was obtained using one-way ANOVA tests (for comparison between 4 groups) or unpaired t tests (comparison between two groups) performed in GraphPad Prism 6 software.

#### 1-Carbon Metabolism Analysis

For bacterial sample collection and metabolite extraction, 3-day old bacterial lawns of BW25113 or mutants grown at 20°C on standard NGM plates were scraped using a 24 cm cell scraper and transferred to an eppendorf tube and snap frozen in liquid nitrogen. The bacterial pellet was kept at −80°C until required.

For worm samples, approximately 1000 synchronized L1 stage larvae were grown at 20°C on standard NGM plates seeded with 3-day old BW25113 or mutant bacteria. L4 larvae were collected for analysis or transferred to fresh NGM plates containing 5-FU and incubated at 25°C for an additional 24 hr before collection. Worms were washed thoroughly using dH_2_O to remove bacteria and snap frozen in liquid nitrogen. The worm pellet was kept at −80°C until required.

For egg samples, approximately 5000 synchronized 1-day adult worms per condition (control and 1 μM 5-FU) were axenized using alkaline hypochlorite. Bleached eggs were placed in M9 buffer for 3 hr to recover from bleaching. An aliquot was kept for determining egg viability, and the remaining eggs were snap frozen in liquid nitrogen and kept −80°C until required.

At least 4 biological replicates from each type of bacteria/worm/egg sample were collected for each measurement. Samples were resuspended in MS sample buffer at pH 7.0 containing 20 mM ammonium acetate, 0.1% ascorbic acid, 0.1% citric acid, 100 mM dithiothreitol (all from Sigma Aldrich). Bacterial suspensions were sonicated for 10 s at 40% amplitude using a hand-held sonicator (Q700 sonicator, Qsonica). Worm and egg pellets were sonicated at 4°C for a total of 1 min 15 s at 100% amplitude. Protein was removed by acetonitrile (Sigma Aldrich) precipitation and centrifugation for 15 min at 12,000 g, 4°C. Supernatants were transferred to fresh tubes, lyophilized, and stored at −80°C until required. Prior to analysis, lyophilized samples were resuspended in 60 μL of MS sample buffer and centrifuged for 15 min at 12,000 g, 4°C. The resulting supernatants were transferred to glass sample vials.

Folate measurements by LC-MS/MS were performed as previously described (Cabreiro et al., 2013). Mass spectrometric data were analyzed as in (Pai et al., 2015) using MassLynx Software (Waters). Statistical analysis was performed by one-way ANOVA using the Graphpad Prism 6 software.

#### Vitamin B_6_ Analysis

For bacterial sample collection and metabolite extraction, 3-day old bacterial lawns grown on standard NGM or supplemented with 1.0 mM pyridoxal at 20°C were washed from plates using 1x PBS. The bacteria were washed three times in 1x PBS to remove potential contaminating pyridoxal from the NGM medium and centrifuged at for 30 min at 4,000 rpm, 4°C. The bacterial pellet was snap frozen in liquid nitrogen and kept at −80°C until required. At least 4 biological replicates were collected for each condition.

The bacterial pellets were resuspended in 50 μL dH_2_O and lysed by subjecting the sample to 5 freeze-thaw cycles using a methanol-dry ice bath and a 37°C waterbath. The resulting lysates were pelleted for 5 min at 5,000 rpm, 4°C, and the supernatants collected. Proteins were precipitated by mixing 10 μL of bacterial lysate supernatant with 0.15 N (final concentration) trichloroacetic acid (TCA, Sigma Aldrich) and spiked with deuterated internal standards (PMP, PNP, PLP: d_3_-PLP, PM: d_3_-PM, PN: d_2_-PN and PL: d_3-_PL; all at a final concentration of 25 nM, Table S1) up to a total volume of 120 μL. Samples were vortexed for 30 s, left on ice in the dark for 60 min and centrifuged to pellet the precipitated protein. The resulting supernatant was transferred to a glass vial and kept at –20°C in the dark until sample analysis.

B_6_ vitamers LC-MS/MS analysis was carried out using an adapted version of the protocol detailed in (Footitt et al., 2013). Stock solutions of all B_6_ vitamers and deuterated internal standards were reconstituted using dH_2_O, stored at –80°C to prevent degradation, and kept on ice protected from light during laboratory handling. A Waters Acquity H-Class UPLC system was connected to a Waters Xevo TQ-S triple quadrupole mass spectrometer in multiple reaction monitoring and positive ionisation mode. A Waters Acquity UPLC HSS T3 column (1.8 μm, 2.1 × 50 mm) with a 1.8 μm Acquity UPLC HSS T3 guard column was used for reversed-phase chromatographic separation along with a mobile phase consisting of A: 3.7% acetic acid (Sigma Aldrich) in H_2_O with 0.02% heptafluorobutyric acid (HFBA, Sigma Aldrich) and B: 100% methanol (Fisher Scientific). A gradient elution over 6.5 min was used. Gradient and retention times of each compound are listed in Table S3. Mass spectrometry settings were as follows: capillary 2.50 kV, source temperature 150°C, desolvation temperature 600°C, cone gas flow rate 150 L/hr, and desolvation gas flow rate 1200 L/hr. The optimized cone voltages and collision energies for each compound are as detailed in Table S3.

Analyte concentrations were determined by taking the peak area ratio of each vitamer and comparing it to a deuterated internal standard (PMP, PNP, PLP compared to d_3_-PLP, PM to d_3_-PM, PN to d_2_-PN and PL to d_3-_PL). All vitamers were well differentiated according to retention time, except for PLP and PNP which elute at a similar time however, these can be differentiated from each other by mass without cross-talk between ion pairs occurring. Calibration curves were constructed between 1.25 and 200 nM and shown to have linearity (r^2^ > 0.99). All results were normalized to protein concentration determined by the Pierce BCA Assay Kit (Thermo Scientific) according to the manufacturer’s instructions. Statistical analysis was performed by one-way ANOVA using GraphPad Prism 6 software.

#### 100 Metabolite Method

For bacterial samples, 3-day old bacterial lawns of BW25113 growing on NGM plates supplemented with 1 μM 5-FU at 20°C plates were washed with dH_2_O, spun down at 15,000 rpm at 4°C and flash frozen in liquid nitrogen. The bacterial pellet was kept at −80°C until required. For worm samples, approximately 4000 synchronized L1 stage larvae were grown at 20°C on NGM plates supplemented 1 μM 5-FU and seeded with 3-day old BW25113. At the L4 larval stage, worms were collected using dH_2_O, washed thoroughly using dH_2_O to remove bacteria, and snap frozen in liquid nitrogen. The worm pellet was kept at −80°C until required. 3-4 biological replicates for bacteria/worm sample were collected for each condition, and an aliquot of each was retained to quantify protein content using the Pierce BCA Assay Kit (Thermo Scientific) for data normalization.

The 100 metabolite analysis was performed as described previously (Nikkanen et al., 2016). Frozen samples were homogenized in two steps protocol for maximum recovery by Precellys-24 bead homogenizer (1.4 mm beads) with 20 μL of labeled internal standard mix (Cambridge Isotope Laboratory). In the first step, 500μL of precooled 100% acetonitrile + 1% formic acid was added to the sample and homogenized for 3 cycles, 20 s each, at 5,500 rpm with 30 s pause between each homogenization interval. Then the samples were centrifuged for 15 min, 14,000 rpm, 4°C and the supernatants were collected. In second step, 500μL of 90/10% acetonitrile/H_2_O + 1% formic acid was added to the remaining pellet, the steps as detailed above were repeated, and supernatants were pooled with the first extract, and the whole extract was centrifuged again for 15 min, 14,000 rpm, 4°C. The collected extracts in both bacterial and worms protocols were dispensed in to OstroTM 96-well plate (Waters Corporation, Milford, USA) and filtered by applying vacuum at a delta pressure of 300-400 mbar for 2.5 min on robot’s (Hamilton’s StarLine) vacuum station. The clean extract was collected to a 96-well collection plate, placed under OstroTM plate. The collection plate was sealed and centrifuged for 15 min, 4000 rpm, 4°C and placed in auto-sampler of the liquid chromatography system for the injection.

100 metabolites were separated by Waters Acquity UPLC, and analyzed by triple quadrupole mass spectrometry. Data analysis was performed using MetaboAnalyst 3.0. Principal component analysis (PCA) using unsupervised multivariate analysis was applied. For each metabolite, differences between experimental groups were determined using linear modeling. Log2 transformed metabolite concentrations with 5-FU were compared against control. Significance of differences was evaluated within the linear models using pooled standard errors with the subsequent Benjamini-Hochberg FDR correction for multiple testing.

#### Nucleotide and Fluoronucleotide Analysis

For bacteria grown on solid media, bacteria were cultured and collected as explained in methods for 1-Carbon metabolism analysis. For bacteria and supernatant analysis, samples were collected as explained in the Bioconversion Assay section and kept at –80°C until further analysis.

For worm samples, approximately 5000 synchronized L1 stage larvae were grown at 20°C on standard NGM plates seeded with 3-day old BW25113 or mutant bacteria. L4 larvae were collected for analysis or transferred to fresh NGM plates containing 5-FU and incubated at 25°C for an additional 8 hr before collection. Worms were washed thoroughly using dH_2_O, frozen in liquid nitrogen and kept at −80°C until further analysis.

3-4 biological replicates from each type of bacteria/worm/supernatant sample were collected for each condition. Each sample was spiked with an optimum concentration of the internal standards (200 μM of Cytidine-^13^C_9_,^15^N_3_ 5′triphosphate and Uridine-^13^C_9_,^15^N_2_ 5′-triphosphate, 10 μM of caffeine, 250 μM of 5-Fluorouridine, Table S1). Bacteria and worm samples were resuspended in 100 μL 100% methanol and sonicated using 30 s intervals at 4°C for a total of 2 min (bacterial samples) or 6 min (worm samples), at 100% amplitude using a high intensity ultrasonic water bath sonicator coupled to a cup horn (Q700 Qsonica). Subsequently, protein was removed by centrifuging lysed samples for 30 min at 13,000 rpm, 4°C and collecting the resulting supernatant to fresh tubes. Samples were lyophilized and the resulting pellet was resuspended in 50 μL of ice-cold dH_2_O prior to sample injection and analysis. Supernatants were filtered through an Amicon Ultra (0.5 mL) centrifugal filter Ultracel 3K (Merck Millipore) for 20 min at 13,000 rpm, 4°C. The resulting filtrate was collected and kept on ice prior to sample injection and analysis.

Nucleotide and fluoronucleotide analysis by LC-MS/MS was performed essentially as described in (Laourdakis et al., 2014) using the ion transitions, cone voltages, and collision energies described in Table S6. Analyte concentrations were determined by correlation of the MS signal of a given metabolite with the appropriate internal standard signal. The results obtained were normalized against protein concentration determined using a Qubit® Fluorometer, according to the manufacturer’s instructions. For each metabolite differences between strains were determined using linear modeling. Log_2_ transformed metabolite concentrations in Δ*ndk,* Δ*yjjG* and Δ*pyrE* mutants were compared against the BW control. Significance of differences was evaluated within the linear models using pooled standard errors.

#### Bacterial Fluorescence Quantification

NGM plates ± pyridoxal (1 mM) were prepared and seeded with 150 μL overnight LB culture of BW25113 strain containing either a promoterless plasmid or the GFP promoter plasmid for *ndk*, *udk*, and *upp* genes (Key Resources Table). The plates were incubated for 3 days at 20°C, then the bacteria were scraped off the plates and washed with 2 mL of 1x PBS into 2 mL eppendorf tubes. The resulting bacterial cultures were thoroughly mixed by vortexing and diluted 4-fold. 75 μL of each sample was added to a 96-well plate containing 75 μL 1x PBS. Bacterial OD was measured at 600 nm and GFP intensity was measured at excitation 470 nm, emission 525 nm using a Tecan Infinite Pro M200 plate reader with Magellan v7.2 software. 4 biological replicates were used for each measurement. Differences between strains were calculated between values determined as: [(GFP gene of interest/OD_600nm_)/GFP promoterless/OD_600nm_]. Statistical analysis was performed by one-way ANOVA using GraphPad Prism 6 software.

#### Bacterial MIC Assays

Bacteria grown overnight in LB-Miller broth were washed twice in NGM broth, normalized to OD_600nm_ = 2 and added to 96-well microtiter plates at a final dilution of 1,000-fold in 200 μL NGM broth containing 2-fold serially diluted drug. Plates were incubated for 16 hr at 37°C, 180 rpm and the absorbance at OD_600nm_ was measured using a Tecan Infinite Pro M200 microplate reader and Magellan v7.2 software. GraphPad Prism 6 was used to create drug response curves and calculate the concentration of drug required for 50% growth inhibition (IC_50_) using a log(inhibitor) versus response - variable slope (four parameter) model, and perform statistical analysis of IC_50_ values by one-way ANOVA.

#### Bioconversion Assays

BW25113 or Keio mutant bacteria were cultured in LB-Miller broth overnight at 37°C. Cultures were spun down at 4000 rpm for 30 min, washed twice with PBS, then resuspended in 1 mL of PBS and the OD_600nm_ determined using a standard spectrophotometer. Each sample was normalized to 12 OD units and centrifuged at 10,000 rpm for 10 min. Bacterial pellets were re-suspended in 1 mL of 5 mM 5-FO or 5-FC in 1X PBS and incubated overnight at 37°C, 650 rpm in the dark. Bacteria were spun down at 10,000 rpm for 5 min, and the resulting supernatants were filter sterilized (0.22 μm) and used for downstream experiments including hatching assays or mass spectrometry.

#### Host-Microbe-Drug Screen

Keio collection bacteria were grown for two generations in LB-Miller broth in 96-well, flat-bottomed microtiter plates incubated for 16 hr at 37°C, 180 rpm, the first passage with 50 μg/mL kanamycin and the second without antibiotics. Screen assay 96-well plates were prepared by mixing 5-FU with NGM agar at the indicated concentrations. Plates were spotted with 5 μL of bacterial culture per well and incubated at 20°C over 3 days to allow bacterial growth. Nematodes were prepared by bleaching gravid adults to release eggs, washing thrice, and synchronizing to L1 in M9 media at 20°C overnight. Approximately 10 L1 nematodes were added per well and incubated at 25°C for 72 hr. Worm development was visually scored to determine minimum inhibitory concentration (MIC, [5-FU] μM) at which worm development was ablated (i.e., to the same degree as worms grown on wild-type BW25113 bacteria at 1 μM 5-FU). Bacterial mutants which displayed no growth on NGM, for which nematode development was delayed in the absence of added drug, or which had undisrupted target gene were excluded from the screen (Table S2). For the primary screen, L1 nematodes on each one of the 3813 knockouts were exposed to 5-FU concentrations (0, 1, 2.5, 5 μM). Mutants with inconsistent results were rescreened. Bacterial clones on which nematodes developed at 5 μM 5-FU (574, 15% of total screened) were further tested in a secondary screen at a wider range of drug concentrations (0, 5, 10, 15, 20, 30, 40, 50, 75, 100 μM) with 3 biological replicates (Figure S2C, right panel). For each mutant, final MIC value was established as the minimum 5-FU concentration that hinders worm growth.

#### Microbe-Drug Screen

Bacterial clones from the worm secondary screen were screened in 5-FU bacterial growth-inhibition assays in 96-well microtiter plates. Mutants were grown in LB for one generation followed by growth in NGM broth for a second generation, before being inoculated in fresh NGM broth for the assay. Plates were incubated at 37°C, 180 rpm, and bacterial growth was measured at 24 hr by OD_600nm_ using a Tecan Infinite Pro M200 plate reader and Magellan v7.2 software. Bacterial OD values were corrected for media only blanks and averaged from 3 independent biological repeats ± SD.

### Quantification and Statistical Analysis

#### General

Data were considered statistically significant when p < 0.05 by unpaired t tests, multiple t tests, one-way ANOVA or Benjamini-Hochberg FDR < 0.05 as indicated in the Figure, Figure labels or experimental methods. Asterisks denote corresponding statistical significance ^∗^p < 0.05; ^∗∗^p < 0.01; ^∗∗∗^p < 0.001. Data are presented as the mean ± SD from at least 3 independent biological replicates, unless stated otherwise in Figures, Figure labels or experimental methods. Statistical analysis was performed using GraphPad Prism 6 software, log-rank test in JMP 11 software (SAS Institute) or linear modeling in R, as indicated.

#### High-Throughput Screens

Data analysis was performed using the R statistical analysis software package v3.2.3 (https://www.r-project.org), unless stated otherwise. Linear modeling/regression was accomplished using R base function “lm,” and function “glm” from “multvar” package. Outliers in linear regression analysis were identified using Bonferonni-adjusted outlier test from “car” package. Plots were generated using “ggplot” and “gplots” packages.

Information on *E. coli* knockouts from the Keio library and their annotations was acquired from (Baba et al., 2006). Additional gene annotations for *E. coli* K-12 were collected using R packages from Bioconductor v3.2: “org.EcK12.eg.db,” “KEGGREST” and “GO.db.” Information on genes with unknown function, enzymes using pyridoxal-5-phosphate (PLP) and metabolic pathways was acquired using PathwayTools and “pythoncyc” Python API software from EcoCyc. All software and databases were used at their most recent versions, as of 2016-08.

The relationship between *E. coli* growth ± 5-FU treatment (OD values) and *C. elegans* (MIC averages) was established using linear regression (Figures S2F and S2G). Outliers Δ*atpH*, Δ*purA*, Δ*priA,* Δ*yfgA*, Δ*hyfF* and Δ*aceE*, with respective Bonferonni-adjusted significance: p = 1.8 × 10^−5^, p = 2.1 × 10^−4^, p = 2.1 × 10^−3^, p = 6.8 × 10^−3^, p = 0.01, p = 0.02 were excluded from the correlation estimation in both datasets. The bacterial growth in control and 5-FU treatment was correlated (Figure S2D). Analysis of knockout and 5-FU interaction in bacteria was performed using linear modeling on log2 transformed growth OD values (Figure S2E). Here, BW25113 (WT) growth without 5-FU was used as reference contrast in corresponding knockout and drug treatment factors. The interaction term shows the difference of combined KO and 5-FU effect from the sum of separate effects. Bacterial strains containing gene deletions that counteract 5-FU effect are considered resistant (antagonistic interaction), whereas strains with gene deletions that strengthen drug effect are considered sensitive (synergistic interaction). The interaction between 5-FU and knockout effect was found to be significantly correlated with the knockout effect itself (y = −0.06-0.35^∗^x, r^2^ = 0.12, p = 2 × 10^−16^, data not shown). This indicates that in general the strains with more impaired growth are more resistant to 5-FU treatment. For example, 5-FU treatment causes 36% growth reduction in WT (OD_Control_ = 0.28, OD_Treatment_ = 0.18) and only 5% in Δ*ldcA*, which grows to ∼31% of WT growth in control (OD_Control_ = 0.08, OD_Treatment_ = 0.07) and has no significant interaction with 5-FU treatment (p = 0.82, FDR = 0.88) (Table S2). The interaction estimates in the linear model were adjusted against this trend to avoid confounding of these effects. The significance of effects in the linear model was estimated using pooled standard errors and Benjamini-Hochberg FDR correction for multiple testing (Table S2).

KEGG and EcoCyc pathway enrichment was estimated in terms of knockout effects on *C. elegans*. Knockouts with MIC values > 5 μM were considered hits and only pathways containing 5 or more corresponding genes were included in the analysis. The pyrimidine pathway was added for illustrative purposes. The significance of the pathway enrichment was calculated using the hypergeometric test, where the p value indicates the likelihood to encounter no less than the observed number of hits in the pathway (right-tail probability), given that the total number of hits is 574 out of 3813 screened knockouts. Coverage was defined as the ratio of hits over the total number of screened knockouts in a specific pathway.

Enrichment of KEGG pathways for compounds from the 100 metabolites study was evaluated using metabolite concentrations and the “Pathway analysis” tool on MetaboAnalyst 3.0.

## Author Contributions

Conceptualization, T.A.S., P.N., and F.C.; Methodology, T.A.S., L.M.Q., P.N., P.P.L., M.P.W., K.-Y.L., L.H.-D., S.S., and F.C.; Software, P.N., A.P., K.B., and V.V.; Formal Analysis, T.A.S., L.M.Q., P.N., P.P.L., M.P.W., L.H.-D., K.B., A.P., V.V., P.B.M., A.T., and F.C.; Investigation, T.A.S., L.M.Q., P.N., P.P.L., M.P.W., K.-Y.L., L.H-D., S.S., and F.C.; Resources, P.C., P.B.M., A.T., N.D.E.G., and F.C.; Writing, T.A.S., L.M.Q., P.N., P.P.L., P.B.M., A.T., N.D.E.G., and F.C.; Visualization, P.N. and F.C.; Supervision, K.B., P.B.M., A.T., N.D.E.G., and F.C.; Project Administration, F.C.; Funding Acquisition, P.C., P.M., A.T., N.D.E.G., and F.C.

## Supplemental Information

Table S1. Statistical Analysis for Hatching Assays, Metabolomics, and a List of Primers, Related to Figures 1 and S1

Table S2. Data for the 5-FU-Microbe-Drug Screen, Including a List of Genes Removed from Analysis and Also Enrichment Analysis Results, Related to Figures 2 and S2

Table S3. Statistical Analysis for Hatching Assays and Vitamin B6 Mass Spectrometry Parameters, Related to Figures 3 and S3

Table S4. Statistical Analysis for Hatching Assays, Related to Figures 4 and S4

Table S5. Statistical Analysis for Hatching Assays and Pyrimidine Mass Spectrometry Parameters, Related to Figures 5 and S5

Table S6. Statistical Analysis for Hatching Assays and Nucleotide Metabolism Data, Related to Figures 6 and S6

Table S7. Statistical Analysis for Hatching Assays and Lifespan Analysis, Related to Figures 7 and S7
